# Tryptophan metabolite norharman secreted by cultivated *Lactobacillus* attenuates acute pancreatitis as an antagonist of histone deacetylases

**DOI:** 10.1186/s12916-023-02997-2

**Published:** 2023-08-28

**Authors:** Qi Zhou, Xufeng Tao, Fangyue Guo, Yu Wu, Dawei Deng, Linlin Lv, Deshi Dong, Dong Shang, Hong Xiang

**Affiliations:** 1 Laboratory of Integrative Medicine, First Affiliated Hospital of Dalian Medical University, No.222 Zhongshan Road, Dalian, 116011 China; 2 Institute (College) of Integrative Medicine, Dalian Medical University, Dalian, 116011 China; 3 Department of Pharmacy, First Affiliated Hospital of Dalian Medical University, Dalian, 116011 China; 4 Department of General Surgery, First Affiliated Hospital of Dalian Medical University, No.222 Zhongshan Road, Dalian, 116011 China

**Keywords:** Acute pancreatitis, *Lactobacillus*, Tryptophan, Norharman, Raftlin 1

## Abstract

**Background:**

Patients with acute pancreatitis (AP) exhibit specific phenotypes of gut microbiota associated with severity. Gut microbiota and host interact primarily through metabolites; regrettably, little is known about their roles in AP biological networks. This study examines how enterobacterial metabolites modulate the innate immune system in AP aggravation.

**Methods:**

In AP, alterations in gut microbiota were detected via microbiomics, and the *Lactobacillus* metabolites of tryptophan were identified by liquid chromatography-tandem mass spectrometry (LC–MS/MS). By culturing *Lactobacillus* with tryptophan, differential metabolites were detected by LC–MS/MS. Lipopolysaccharide (LPS)-stimulated RAW264.7 cells and mice with cerulein plus LPS-induced AP were used to evaluate the biological effect of norharman on M1 macrophages activation in AP development. Further, RNA sequencing and lipid metabolomics were used for screening the therapeutic targets and pathways of norharman. Confocal microscopy assay was used to detect the structure of lipid rafts. Molecular docking was applied to predict the interaction between norharman and HDACs. Luciferase reporter assays and chromatin immunoprecipitation (ChIP) were used to explore the direct mechanism of norharman promoting *Rftn1* expression. In addition, myeloid-specific *Rftn1* knockout mice were used to verify the role of *Rftn1* and the reversed effect of norharman.

**Results:**

AP induced the dysfunction of gut microbiota and their metabolites, resulting in the suppression of *Lactobacillus*-mediated tryptophan metabolism pathway. The *Lactobacillus* metabolites of tryptophan, norharman, inhibited the release of inflammatory factor in vitro and in vivo, as a result of its optimal inhibitory action on M1 macrophages. Moreover, norharman blocked multiple inflammatory responses in AP exacerbation due to its ability to maintain the integrity of lipid rafts and restore the dysfunction of lipid metabolism. The mechanism of norharman’s activity involved inhibiting the enzyme activity of histone deacetylase (HDACs) to increase histone H3 at lysine 9/14 (H3K9/14) acetylation, which increased the transcription level of *Rftn1* (Raftlin 1) to inhibit M1 macrophages’ activation.

**Conclusions:**

The enterobacterial metabolite norharman can decrease HDACs activity to increase H3K9/14 acetylation of *Rftn1*, which inhibits M1 macrophage activation and restores the balance of lipid metabolism to relieve multiple inflammatory responses. Therefore, norharman may be a promising prodrug to block AP aggravation.

**Graphical Abstract:**

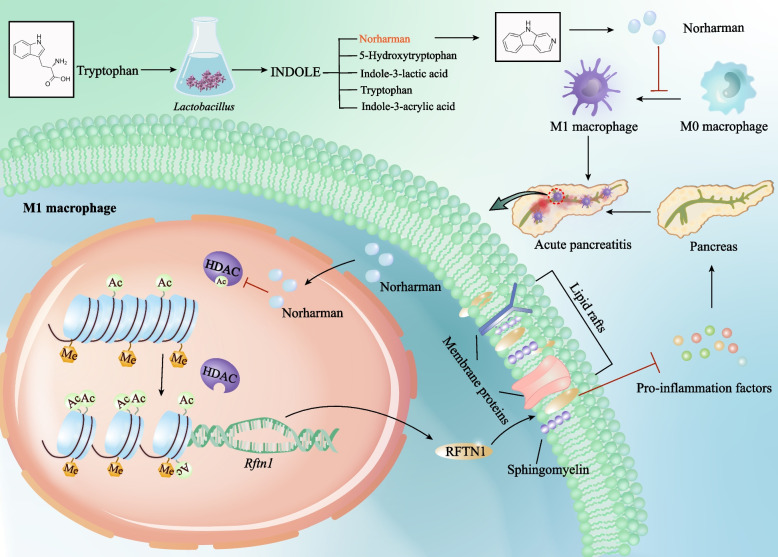

**Supplementary Information:**

The online version contains supplementary material available at 10.1186/s12916-023-02997-2.

## Background

Acute pancreatitis (AP) remains a leading cause of emergency hospitalization for gastrointestinal conditions [1]. In particular, severe cases ending in infectious complications and organ failure are potentially lethal, with mortality rates up to 20–40% [2]. According to convincing evidence and our previous study, AP has a particular gut microbial phenotype, characterized by a decrease in microbial diversity and an increase in pathogenic bacterial abundance [3, 4]. A significant higher severity rate of mild AP was observed for patients with altered gut microbiota compared with patients with unchanged gut microbiota [5]. In vivo, depleting the gut microbiota by establishing antibiotic-treated mice and germ-free mouse models alleviated pancreatic injury after AP induction. Conversely, recolonization of the bacteria from specific pathogen-free (SPF) mice by fecal microbiota transplantation exacerbated AP [6]. Hitherto, how gut microbiota regulates AP exacerbation is not fully elaborated.

The intermediates or end products of microbial metabolism are one of the primary modes by which gut microbiota crosstalk with the host [7]. In recent years, the interaction between microbial metabolites and host immunity has attracted increased interest. Gut microbiota catabolizes carbohydrates, proteins, fats, and vitamins into functional products, such as short-chain fatty acids, bile acids, and tryptophan metabolites, and the latter is extensively involved in host immune homeostasis [8, 9]. Studies have found that many circulating metabolites can only be detected in the presence of gut microbiota, especially tryptophan indole metabolites [10]. Although active metabolites with regulatory functions in cell signaling continue to be discovered, only a few microbial metabolites have been shown to modulate specific immune parameters [11]. The regulatory effect of bacterial metabolites on host immunity is still a treasure house to be excavated.

AP starts with sterile local inflammation and then possibly progresses to systemic inflammatory response syndrome (SIRS) induced by the infiltration of inflammatory cells into the local milieu, followed by compensatory anti-inflammatory response syndrome (CARS) [12]. A proinflammatory/antiinflammatory cytokines imbalance destroys host immunity, causing AP to be severe [13]. Macrophages are reported as scavengers that regulate the immune response against pathogens in tissue inflammation, injury, and repair processes [14]. Infiltrating macrophages with high plasticity can be polarized into classically activated macrophages (M1 macrophages) stimulated by LPS and release proinflammatory cytokines (such as tumor necrosis factor-alpha (*Tnf-α*)*,* interleukin-1beta (*Il-1β*), and interleukin-6 (*Il-6*)), which markedly aggravate SIRS caused by AP [15]. We previously identified macrophage infiltration and activation as a “trigger event” to initiate AP severity, and this process may be a promising target for AP immunotherapy [16]. Recent studies have revealed that gut microbial metabolites skew macrophage polarization and exert a marked impact on disease development [17]. For example, fecal deoxycholic acid produced by gram-positive bacteria dose-dependently promoted M1 macrophage polarization and proinflammatory cytokine production in colonic inflammation [18]. However, there is a lack of research focusing on how the gut microbiota and its metabolites modulate macrophage polarization to progressively aggravate AP.

In this study, we demonstrated the dramatic disturbance of *Lactobacillus*-mediated tryptophan metabolism during AP. We then elucidated the roles of enterobacterial metabolites in M1 macrophage activation and the underlying mechanism to relieve AP aggravation. It raised the possibility that treatment with enterobacterial metabolites is a promising approach to block macrophage-induced AP aggravation.

## Methods

### Animals

Sprague–Dawley (SD) rats and wild-type (WT) C57BL/6 mice were purchased from the Experimental Animal Center of Dalian Medical University (Dalian, China). *Rftn1*[flox/flox, lyz2-cre] mice were purchased from Cyagen Biosciences, Inc. (Guangzhou, China). All experimental procedures were conducted in compliance with the People’s Republic of China Legislation Regarding the Use and Care of Laboratory Animals. We constructed rat and mouse AP models as previously reported [19, 20].

### 16S rDNA sequencing

After DNA extraction and PCR amplification, the samples were sequenced on an Illumina MiSeq platform according to our previous method [3].

### Chromatin immunoprecipitation (ChIP) assay

ChIP assays were performed using a ChIP kit (CST#9003, USA) according to the manufacturer’s instructions. Eluted purified DNA was used for quantitative reverse transcription (qPCR) assays by specific primers (Additional file 1: Table S1).

### Others

Additional experimental details including AP model establishment, bacterial culture, LC–MS/MS analysis, cell viability and cytotoxicity, cell transfection, RNA sequencing, confocal microscopy, lipid metabolomics, luciferase assay, molecular docking, chromatin immunoprecipitation (ChIP) assay, ELISA, isolation of bone marrow-derived macrophages (BMDMs), infection of BMDMs with recombinant Rftn1 lentiviral vector, agarose gel electrophoresis, serum enzyme assays, hematoxylin and eosin and double-color immunofluorescence staining, flow cytometry analysis, qPCR, western blotting, and statistical analysis are provided in the Supplementary Information.

## Results

### Pathway of *Lactobacillus*-mediated tryptophan metabolism was obviously inhibited in AP model

**Fig. 1:**
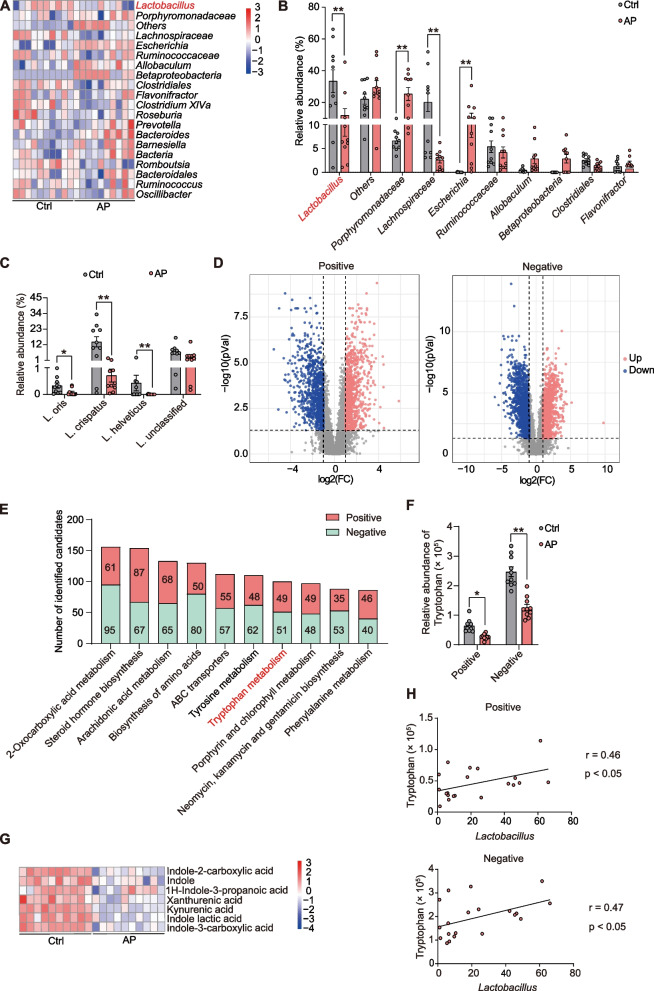
Pathway of *Lactobacillus*-mediated tryptophan metabolism was obviously inhibited in AP model. **A** Gut microbiota imbalance in rat feces of the AP group compared with the Ctrl group based on 16S rDNA sequencing (*n* = 10). **B** Alterations in the gut microbiota between the Ctrl and AP groups (*n* = 10). **C** The relative abundance of *Lactobacillus* species in rat feces was decreased in the AP group compared with the Ctrl group (*n* = 10). **D** Metabolite disorder was shown in the feces of the rats in the AP group compared with the Ctrl group based on untargeted metabolomics (*n* = 10). **E** The differentially abundant metabolites were enriched in various metabolic pathways. **F** The relative abundance of tryptophan was significantly reduced in feces of the AP group compared with the Ctrl group (*n* = 10). **G** Both endogenous and gut microbiota tryptophan metabolism were indeed inhibited in AP. **H** The abundance of *Lactobacillus* was positively correlated with the level of tryptophan (*n* = 10). Data are presented as the mean ± SEM; *P < 0.05 and **P < 0.01 vs. the Ctrl group

Gut microbiota and host metabolism have been a research hotspot in recent years. To explain the relationship of AP-induced metabolism disorder, we performed 16S rDNA gene sequencing and untargeted metabolomics of fresh fecal samples obtained from rats with AP, and sham operation was used as a control. Consistent with previous research, AP has a characteristic gut microbiota, mainly manifested as a decrease in beneficial bacteria and an increase in pathogenic bacteria (Fig. 1A, B). The relative abundance of *Lactobacillus* species, including *Lactobacillus oris*, *Lactobacillus crispatus*, and *Lactobacillus helveticus*, was decreased in the AP group compared with the Ctrl group (Fig. 1C). According to the results of untargeted metabolomics, we found 798 metabolites were upregulated but 826 metabolites were downregulated in positive model, and 1117 metabolites were upregulated but 1207 metabolites were downregulated in negative model (Fig. 1D). This result indicated metabolite disorder was observed in the feces from AP. As shown in Fig. 1E, the differentially abundant metabolites were significantly enriched in multiple metabolic pathways. Literature review revealed that gut *Lactobacillus*-mediated tryptophan metabolism modulated host susceptibility and disease development [21, 22]. Particularly, the relative abundance of tryptophan was absolutely downregulated in the AP group compared with the Ctrl group (Fig. 1F). Tryptophan was regarded as an essential substance in host physiology, and the three tryptophan metabolism pathways were regulated by gut microbiota, involving 5-hydroxytryptamine (5-HT) production, ligands of the aryl hydrocarbon receptor (AhR), and kynurenine-producing indoleamine 2,3-dioxygenase pathways [23]. Moreover, metabolites of kynurenine pathway (kynurenic acid and xanthurenic acid) and indole and indole derivatives were declined in the AP group, suggesting both endogenous and gut microbiota tryptophan metabolism were indeed inhibited in AP (Fig. 1G). We speculated that *Lactobacillus*-mediated tryptophan metabolism may play a central role in AP. To further verify the relationship between *Lactobacillus* and tryptophan metabolism. We conducted the correlation analysis and confirmed that the abundance of *Lactobacillus* was positively correlated with the level of tryptophan in both negative and positive models (Fig. 1H), indicating the dysfunction of tryptophan metabolism occurs in microbiota-host crosstalk in AP. Taken together, these findings indicated that AP induced gut microbiota dysfunction and metabolic imbalance, resulting in the suppression of *Lactobacillus*-mediated tryptophan metabolism pathway.

### Cultivated *Lactobacillus* secreted tryptophan metabolite norharman, which inhibited M1 macrophage activation in vitro

**Fig. 2:**
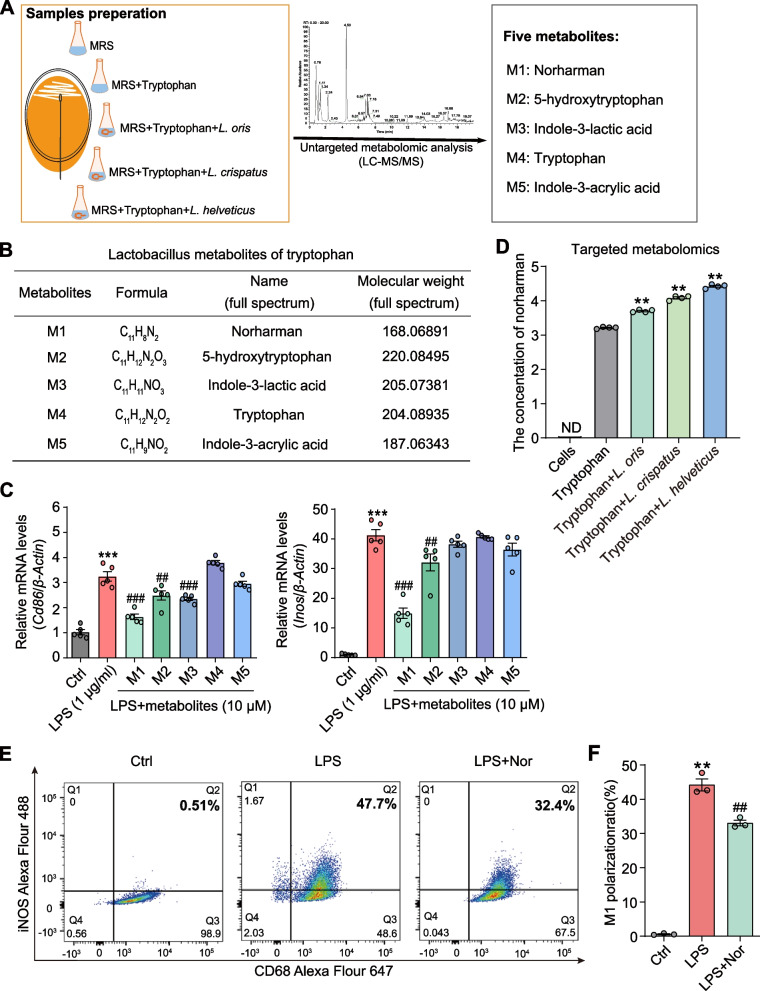
Cultivated *Lactobacillus* secreted tryptophan metabolite norharman, which inhibited macrophage M1 activation in vitro.** A** Schematic diagram of LC–MS/MS. **B** A total of five *Lactobacillus* metabolites of tryptophan were identified by applying LC–MS/MS. **C** The effects of 5 metabolites on M1 macrophages were detected and norharman showed the strongest inhibitory effect by using qPCR (*n* = 5). **D** Norharman was mainly from tryptophan metabolism regulated by *Lactobacillus* by targeted LC–MS/MS (*n* = 4). **E**, **F** Norharman inhibited the activation of M1 macrophages, as shown by flow cytometry analysis (*n* = 3). Data are presented as the mean ± SEM; **P < 0.01 vs. the Ctrl group or tryptophan group; ^##^*P* < 0.01 vs. the LPS group

Although various studies have expounded the relationship between gut microbiota dysfunction and metabolic imbalance in AP, there is a lack of in-depth mechanistic research focusing on *Lactobacillus*-mediated tryptophan metabolism in AP. Based on AP-induced decreases in the abundance of *Lactobacillus* and tryptophan, we cultured *Lactobacillus* with tryptophan to further clarify how *Lactobacillus* regulates tryptophan metabolism. A total of three *Lactobacillus* strains, *L. oris*, *L. crispatus*, and *L. helveticus*, were cultured in tryptophan-rich medium in vitro, and a schematic diagram of LC–MS/MS was displayed in Fig. 2A. Absorbance detection showed that several low concentrations of tryptophan could not promote the proliferation of *Lactobacillus* strains, but *Lactobacillus* growth was significantly inhibited by 1% tryptophan (Additional file 1: Fig. S1A). Thus, *Lactobacillus* strains were cultured in a medium with 0.1% tryptophan for the detection of differential metabolites. As shown in Fig. 2B, a total of five *Lactobacillus* metabolites of tryptophan were identified as indoles and their derivatives, including norharman, tryptophan, 5-HT, indole-3-lactic acid and indole-3-acrylic acid, based on both negative and positive models of LC–MS/MS.

Regarding classically activated (M1) macrophages exhibiting proinflammatory phenotypes in AP, we explored the roles of five *Lactobacillus* metabolites in macrophage activation. LPS is a classic inducer of M1 macrophages in vitro, and RAW264.7 cells were stimulated with three increasing concentrations of LPS: 0.1, 1, and 10 μg/ml. Then, LPS at 1 μg/ml was validated as the optimal concentration for M1 macrophage activation by qPCR and flow cytometry (Additional file 1: Fig. S1B-C). In addition, RAW264.7 cells were treated with above five metabolites for 24 h to evaluate the cytotoxicity, and finally, 10 μM was determined to be the maximum nontoxic concentration in vitro (Additional file 1: Fig. S1D). Interestingly, two metabolites (norharman and 5-HT) could inhibit macrophage M1 polarization, as shown by the detection of *Cd86* and inducible nitric oxide synthase (*iNOS)* expression (markers of M1 macrophages), and norharman had the strongest inhibitory effect (Fig. 2C). To explore whether norharman was exogenous or endogenous, we examined the level of norharman in RAW264.7 cells and culture supernatant of tryptophan broth medium with or without *Lactobacillus* strains by targeted LC–MS/MS. The results showed that there was no intracellular norharman in RAW264.7 cells, whereas the concentration of norharman was increased in the culture supernatant of tryptophan broth medium with *Lactobacillus* strains compared with those without *Lactobacillus* strains, suggesting that norharman was mainly from tryptophan metabolism regulated by *Lactobacillus* (Fig. 2D). Norharman also significantly reduced the M1 macrophage ratio after LPS stimulation, as shown by flow cytometry (Fig. 2E, F).

These data suggested that tryptophan metabolites could suppress M1 macrophage activation, and norharman, which was exogenous and mainly generated from tryptophan metabolism regulated by *Lactobacillus*, had the strongest inhibitory effect.

### Norharman reduced macrophage M1 activation to reverse the pathological phenotype in mice with AP

**Fig. 3:**
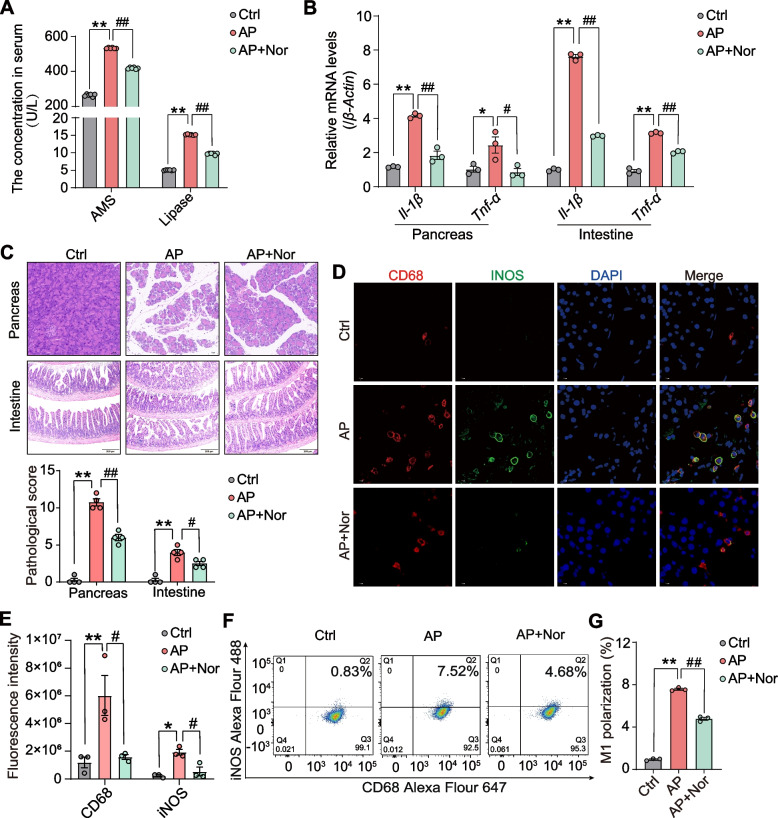
Norharman reduced M1 macrophage activation to reverse the pathological phenotype in mice with AP.** A** Norharman decreased the levels of serum amylase and lipase in the mice with AP (*n* = 6). **B** Norharman downregulated the mRNA expression of the proinflammatory cytokines *Tnf-α* and *Il-1β* by qPCR (*n* = 3). **C** Norharman reversed AP-induced pancreatic and intestinal damage in mice by HE staining (*n* = 4). **D**, **E** Norharman suppressed M1 macrophage activation of the pancreas in the mice with AP, as shown by immunofluorescence (*n* = 3). **F**, **G** Norharman reduced the ratio of M1 macrophages in the spleen of the mice with AP by flow cytometric analysis (*n* = 3). Data are presented as the mean ± SEM; *P < 0.05 and **P < 0.01 vs. the Ctrl group; ^#^*P* < 0.05 and ^##^*P* < 0.01 vs. the AP group

On the basis of significant inhibition of M1 macrophage activation in vitro, we also validated the effect of the enterobacterial metabolite norharman in suppressing macrophage M1 activation in AP mouse models. Toxicological studies showed that the serum levels of pancreatic toxicity, hepatotoxicity, and nephrotoxicity markers, including AMS, ALT and AST, BUN, and CRE, were unaffected by increasing doses of norharman in mice (Additional file 1: Fig. S2A-E). Thus, the maximum dose of norharman (100 mg/kg for 24 h) was chosen for the pharmacodynamics experiments to confirm the effect of norharman in vivo. As shown in Fig. 3A, B, the levels of serum AMS and lipase, as well as the mRNA expression of *Tnf-α* and *Il-1β* in pancreatic and intestinal tissue, were significantly higher in the AP group than that in Ctrl group but markedly decreased after norharman treatment. HE staining demonstrated obvious pancreatic edema, inflammatory infiltration, and destruction of intestinal villi in the AP group compared with the Ctrl group. However, norharman reduced pancreatic lesions and inflammation, maintained the integrity of intestinal villi structure, and reduced intestinal damage (Fig. 3C). As shown in Fig. 3D, E, an increased number of CD68^+^/iNOS^+^ M1 macrophages (red and green fluorescent regions) was observed in the pancreas from the AP group compared to the Ctrl group, but the fluorescence intensity was obviously reduced in the norharman group. Flow cytometric analysis of the spleen presented similar results. Compared with the Ctrl mice, the mice with AP had a higher M1 macrophage ratio, which was decreased after norharman treatment (Fig. 3F, G). These results confirmed that norharman ameliorated the pathology of the mice with AP by inhibiting M1 macrophage activation.

### Norharman targeted Raftlin 1 (Rftn1) to restrain M1 polarization of macrophages

**Fig. 4:**
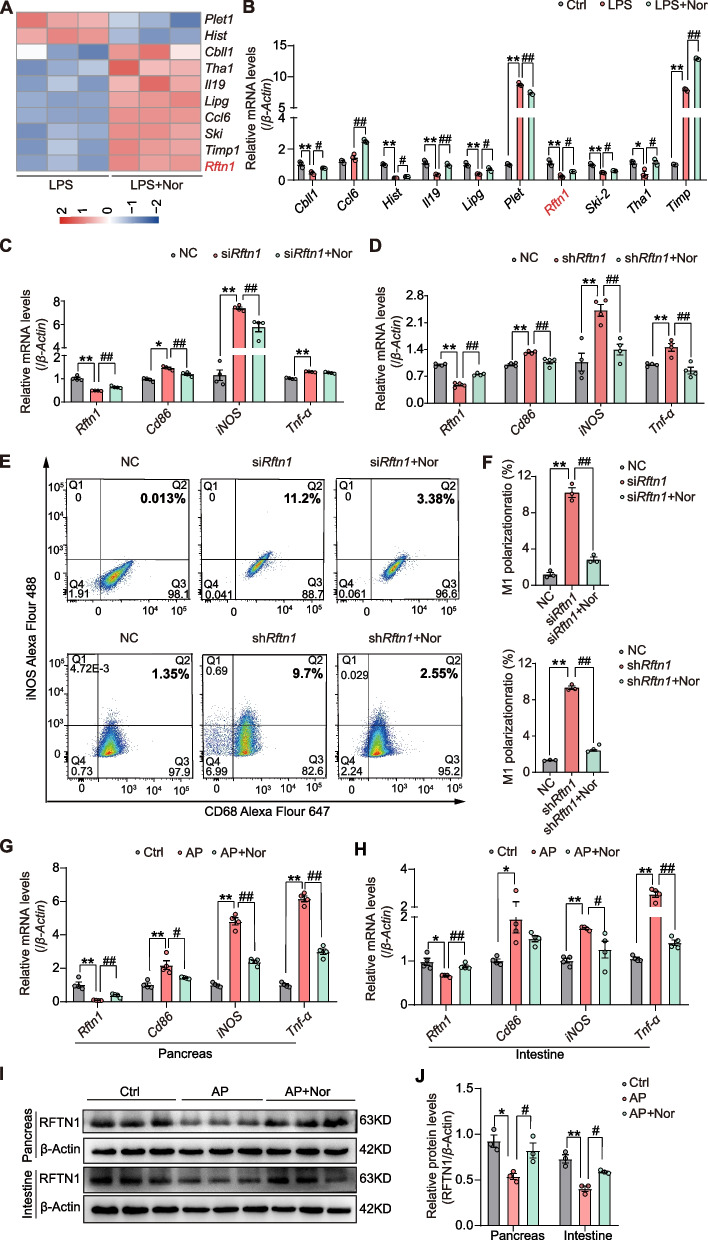
Norharman targeted *Raftlin 1* (*Rftn1*) to restrain the M1 polarization of macrophages. **A** Heatmap of RNA sequences (*n* = 3). **B** Validation of DEGs in vitro by qPCR (*n* = 3). **C**, **D** Norharman inhibited M1 macrophage activation after *Rftn1* knockdown by qPCR (*n* = 4). **E**, **F** Norharman reversed the high ratio of M1 macrophages induced by *Rftn1* knockdown through flow cytometric analysis (*n* = 3). **G**, **H** Norharman upregulated the mRNA expression of *Rftn1* and M1 macrophage markers in pancreatic and intestinal tissues of the mice with AP by qPCR and western blot (*n* = 4). **I**, **J** Norharman upregulated the protein expression of *Rftn1* in pancreatic and intestinal tissues of the mice with AP by western blot (*n* = 3). Data are presented as the mean ± SEM; *P < 0.05 and **P < 0.01 vs. the NC or Ctrl group; ^#^*P* < 0.05 and ^##^*P* < 0.01 vs. the si*Rftn1* or sh*Rftn1* or AP group

To further investigate the targets of norharman that regulate macrophage polarization, we used RNA sequencing to analyze gene alterations at the transcriptome level. The heatmap in Fig. 4A shows the top 10 DEGs between the LPS group and the LPS + Nor group, including placenta-expressed transcript 1 (*Plet1*), histone cluster 1 (*Hist*), casitas B-lineage proto-oncogene like 1 (*Cbll1*), threonine aldolase (*Tha1*), interleukin-19 (*Il-19*), endothelial lipase (*Lipg*), chemokine (C–C motif) ligand 6 (*Ccl6*), ski sarcoma viral oncogene homolog (*Ski*)*,* tissue inhibitor of metalloproteinase 1 (*Timp1*) and *Rftn1*; these genes were verified by qPCR in vitro (Fig. 4B). According to a literature review, we found that *Rftn1* encodes the Raftlin protein, which can control clathrin-associated adaptor protein-2 in clathrin-mediated endocytosis of TLR4 ligands in macrophages, as well as modulate cell entry of poly(I:C), which is important for activation of TLR3 in human myeloid dendritic cells and epithelial cells [24, 25].

To determine whether *Rftn1*-mediated macrophage polarization, we designed *Rftn1* knockdown by using siRNA and shRNA to detect the change in polarization phenotype. After transfection of three siRNAs/shRNAs into RAW264.7 cells, the siRNA/shRNA with the best knockdown efficiency was chosen for the follow-up studies (Additional file 1: Fig. S3A-B). Compared with the NC treatment, knockdown of *Rftn1* increased the mRNA expression of M1 macrophage markers, whereas norharman decreased the mRNA expression of M1 macrophage markers by upregulating *Rftn1* expression (Fig. 4C, D). Moreover, the *Rftn1* knockdown groups had a higher ratio of M1 macrophages than the NC group, which was significantly reduced in the si*Rftn1*/sh*Rftn1* + Nor group (Fig. 4E, F). As shown in Fig. 4G, H, the mRNA expression of *Rftn1* and M1 macrophage markers was increased in the AP group compared with that in the Ctrl group, whereas decreased in the AP + Nor group, except the mRNA expression of *Cd86* without significant difference. In addition, the protein levels of *Rftn1* in the pancreas and intestine were significantly decreased in the AP group compared with the Ctrl group, whereas they were increased in the AP + Nor group (Fig. 4I, J). Furthermore, we have examined the change of intestinal tight junction proteins (Claudin-1, Occludin-1 and ZO-1), and protective mucins (Mucin-2). As shown in Additional file 1: Fig. S4, the expression of intestinal tight junction proteins (Claudin-1, Occludin-1, and ZO-1) was decreased in the AP group compared with the Ctrl group, which was increased in the AP + nor group. Similarly, we observed less expression of protective mucins (Mucin-2) in the AP groups compared with that in the Ctrl group, and norharman could reverse this phenotype. These results revealed that *Rftn1* is a crucial regulator of norharman in restraining the M1 polarization of macrophages.

### Norharman restored the disruption of lipid rafts and lipid metabolism by inhibiting multiple inflammatory responses after Rftn1 knockdown

**Fig. 5:**
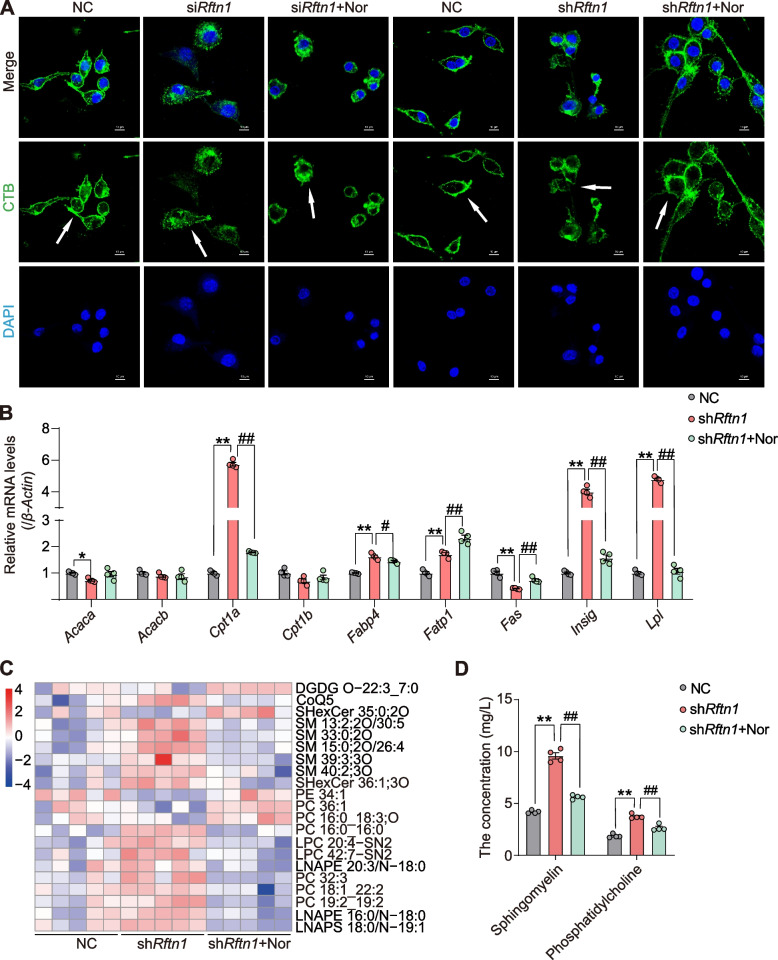
Norharman restored the disruption of lipid rafts and lipid metabolism after *Rftn1* knockdown.** A** Norharman alleviated the structural disruption of lipid rafts induced by *Rftn1* knockdown by laser confocal microscopy (1000 ×). **B** Alterations in lipid-related genes by qPCR (*n* = 5). **C** The lipidomic heatmap (*n* = 5). **D** The levels of sphingomyelin (SM) and phosphatidylcholine (PC) were validated in vitro by using commercial kits (*n* = 4). Data are presented as the mean ± SEM; *P < 0.05 and **P < 0.01 vs. the NC group; ^#^*P* < 0.05 and ^##^*P* < 0.01 vs. the si*Rftn1* or sh*Rftn1* group

As a novel major protein in lipid rafts, Raftlin is localized exclusively in lipid rafts by fatty acylation of N-terminal Gly2 and Cys3, influencing the structural integrity of lipid rafts and participating in lipid metabolism [26]. To determine the structure of lipid rafts affected by *Rftn1*, we used cholera toxin B (CTB) as a classic marker of membrane lipid rafts as previously reported [27, 28]. As shown in Fig. 5A, CTB-positive macrophages appeared round or fusiform in shape with dense fluorescence in the NC group by laser confocal microscopy. However, the destruction of lipid rafts structures characterized by scattered fluorescence was observed following si*Rftn1*/sh*Rftn1* transfection in vitro, but this effect was reversed by norharman treatment.

Based on these results, we hypothesized that *Rftn1* may influence lipid metabolism. Therefore, we detected the expression of lipid metabolism-related genes and notably found that *Rftn1* knockdown affected the expression of lipid metabolism-related genes, including acetyl coenzyme A carboxylase alpha (*Acaca*), acetyl coenzyme A carboxylase beta (*Acacb*), carnitine palmitoyltransferase 1a (*Cpt1a*), carnitine palmitoyltransferase 1b (*Cpt1b*), fatty acid binding protein 4 (*Fabp4*), fatty acid transport protein 1 (*Fatp1*), fatty acid synthase (*Fas*), insulin-induced gene (*Insig*), and lipoprotein lipase (*Lpl*) (Fig. 5B, Additional file 1: Fig. S5). Subsequently, lipid metabolomics further confirmed the imbalance of lipid metabolism in macrophages after *Rftn1* knockdown, which mainly manifested as increased production of sphingomyelin (SM) and phosphatidylcholine (PC), but norharman treatment reversed these phenotypes (Fig. 5C, D). Recent research has suggested that SM protects against dysfunctional lipid metabolism, gut dysbiosis, antigen presentation, and inflammation [29, 30]. At present, the anti-inflammatory properties of PC have attracted increased attention. PC could benefit systemic inflammation via the gut-brain axis [31], decrease the expression of other lipases and the lipogenic marker peroxisome proliferator-activated receptor-γ, and induce lipolysis in adipose tissue [32]. Together, these findings indicated that norharman could inhibit the M1 polarization of macrophages by restoring the imbalance of lipid metabolism.

**Fig. 6:**
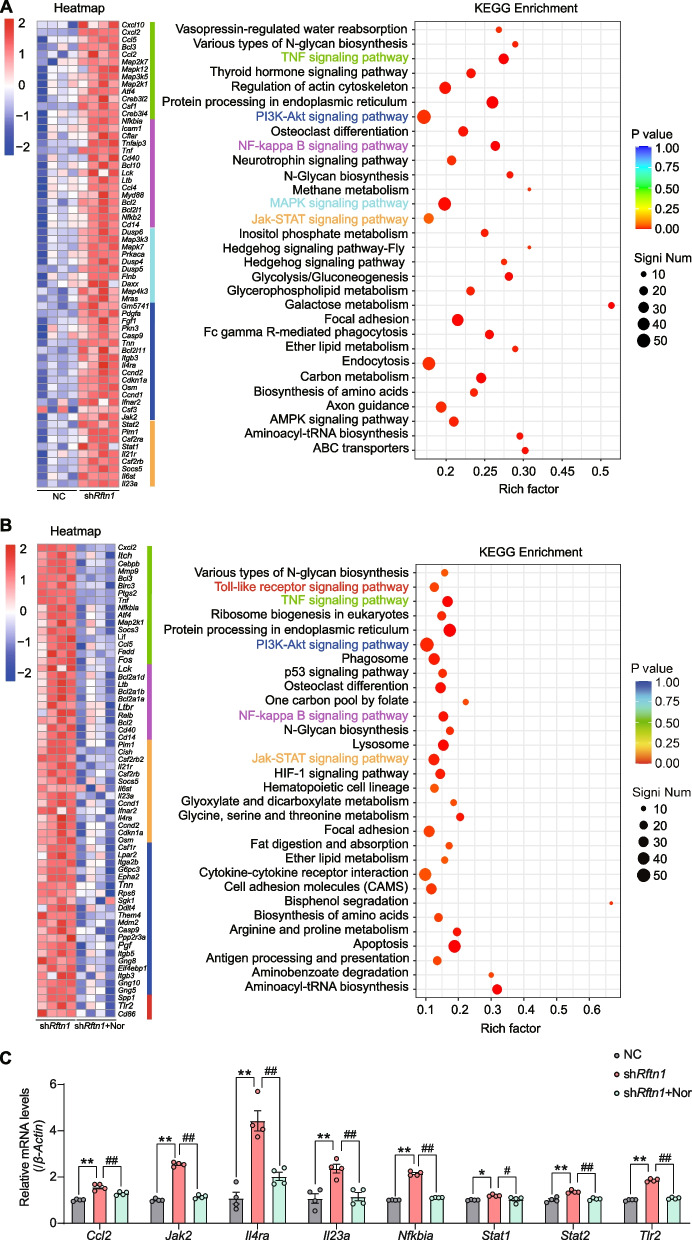
Norharman restrained the polarization of M1 macrophages by inhibiting multiple proinflammatory responses. **A** Heatmap of RNA-seq and KEGG enrichment analyses showed that multiple inflammatory pathways were activated in sh*Rftn1* group (*n* = 4). **B** Heatmap of RNA-seq and KEGG enrichment analyses showed that multiple inflammatory pathways were inhibited in the sh*Rftn1* + Nor group (*n* = 4). **C** Validation of inflammation-related genes in vitro by qPCR (*n* = 4). Data are presented as the mean ± SEM; *P < 0.05 and **P < 0.01 vs. the NC group; ^#^*P* < 0.05 and ^##^*P* < 0.01 vs. the sh*Rftn1* group

Given the strong relationship between lipid metabolism and the inflammatory response in macrophage polarization, we applied RNA sequencing to identify the key candidate gene changes and analyzed downstream inflammatory signaling pathways after *Rftn1* knockdown and norharman treatment. We found that various proinflammatory factors were upregulated in the sh*Rftn1* group, including C-X-C motif ligand 2 (*Cxcl2*), C-X-C motif ligand 10 (*Cxcl10*), chemokine (C–C motif) ligand 2 (*Ccl2*), myeloid differentiation primary response 88 *(Myd88)*, janus kinase 2 (*Jak2*), and interleukin-23a (*Il23a*). In contrast, the expression of proinflammatory factors, such as *Cxcl2*, *Il23a*, *Tnf*, and toll-like receptor 2 (*Tlr2*), was downregulated in the shRftn1 + Nor group. Furthermore, KEGG enrichment analysis revealed that norharman could block multiple pathways related to the inflammatory response induced by *Rftn1* knockdown, such as the TNF signaling pathway, NF-κB pathways, MAPK signaling pathway, PI3K signaling pathway, and JAK-STAT signaling pathway (Fig. 6A, B). To validate these DEGs in vitro, we examined the mRNA expression levels of NF-kappaB (*Nfkbia*), signal transducer and activator of transcription 1 and 2 (*Stat1* and *2*), *Ccl2*, *Jak2*, *Tlr2*, *Cxcl2*, *Il23a*, and interleukin-4 receptor alpha (*Il4ra*), which were consistent with the RNA sequencing results (Fig. 6C).

These results suggested that norharman could promote *Rftn1* expression to inhibit macrophage M1 polarization and restore the reprogramming of lipid metabolism, which blocked various proinflammatory responses.

### Norharman promoted Rftn1 transcription by inhibiting the enzymatic activity of HDACs to increase H3K9/14 acetylation

**Fig. 7:**
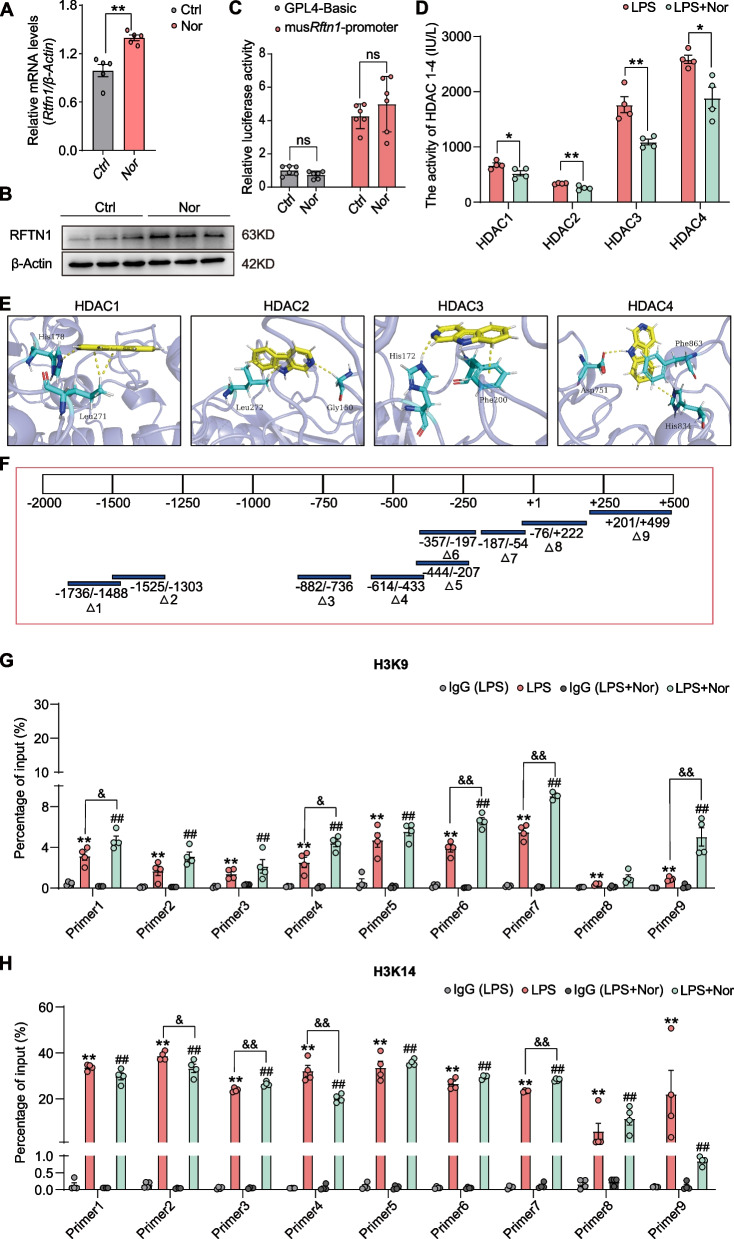
Norharman promoted *Rftn1* transcription by inhibiting the enzymatic activity of HDACs to increase H3K9/14 acetylation.** A**, **B** Norharman significantly increased *Rftn1* mRNA and protein expression by qPCR and western blot (*n* = 5). **C** Norharman did not bind directly to the *Rftn1* promoter region, as verified by a dual-luciferase reporter assay (*n* = 6). **D** Norharman significantly inhibited the enzymatic activity of HDAC1-4 by measuring the enzymatic activity of HDAC1-4 (*n* = 4). **E** The detailed 3D binding mode of HDAC1-4 with norharman by molecular docking analysis. The compound is colored yellow, the surrounding residues in the binding pockets are colored cyan, and the backbone of the receptor is depicted as a slate cartoon. The hydrogen bonds are depicted as yellow dashed lines. **F** The *Rftn1* promoter region (− 2000/ + 500) was truncated into 9 segments. **G**, **H** Norharman increased active H3K9/14Ac signals at the *Rftn1* promoter by ChIP assay (*n* = 4). Data are presented as the mean ± SEM; *P < 0.05 and **P < 0.01 vs. the IgG (LPS), Ctrl or LPS group; ^#^*P* < 0.05 and ^##^*P* < 0.01 vs. the IgG (LPS + Nor) group; ^&^*P* < 0.05 and ^&&^*P* < 0.01 vs. the LPS group

Inspired by the above data, we determined that norharman inhibited M1 macrophage activation by promoting *Rftn1* expression, and we also validated the mRNA and protein levels of *Rftn1* after norharman treatment. The results showed that norharman could significantly upregulate *Rftn1* expression at both the mRNA and protein levels in RAW264.7 cells (Fig. 7A, B). To gain insight into the molecular mechanisms by which norharman promoted *Rftn1* expression, we hypothesized that norharman could directly bind to the *Rftn1* promoter region and promote its transcription, and we performed dual-luciferase reporter assays. As shown in Fig. 7C, HEK293T cells were transfected with a luciferase plasmid containing the sequence of the *Rftn1* promoter or control sequence, and we found that norharman could not increase *Rftn1* promoter-driven luciferase activity, suggesting that norharman promoted *Rftn1* transcription not by directly binding to its promoter region but through other regulatory means.

Through a literature review, we found that the norharman analog β-carboline tethered cinnamoyl 2-aminobenzamides could serve as class I selective HDAC inhibitors, and a series of novel β-carboline-based hydroxamate derivatives exhibited profound HDAC1/3/6 inhibitory effects [33, 34]. In addition, acetylation on histones is regulated by histone acetyltransferases, while HDACs can remove acetyl groups from the N-terminal tail of histones [35]. To verify the relationships between norharman and HDACs, we measured the enzymatic activity of HDAC1-4 after norharman treatment and found that norharman could inhibit the enzymatic activity of HDAC1-4 (Fig. 7D).

Subsequently, molecular docking analysis was used to confirm the interactions of HDAC1-4 and norharman. As a result, the docking scores (Kcal/mol) of HDAC1-4 showed a decreasing trend; thus, HDAC4 possessed the best binding ability with the norharman compounds. The representative three-dimensional patterns of docking combinations are shown in Fig. 7E. To determine whether H3K9/14 acetylation was recruited directly to the *Rftn1* gene, we performed ChIP and qPCR analyses of RAW264.7 cells. For determination of the specific binding sites, the *Rftn1* promoter region (− 2000/ + 500) was truncated into 9 segments (Fig. 7F, Additional file 1: Table S1). ChIP experiments showed enrichment of H3K9/14ac signal binding around all elements of the *Rftn1* promoter in the LPS group, while increased H3K9 acetylation signals were also enriched around special elements (− 1736/ − 1488, − 614/ − 433, − 357/ − 54, and + 201/ + 499 bp) after norharman treatment, and increased H3K14 acetylation signals enriched around (− 882/ − 736 and − 186/ − 54 bp) after norharman treatment (Fig. 7G, H). Taken together, these findings demonstrated that norharman promoted *Rftn1* transcription by repressing the activity of HDACs to increase H3K9/14 acetylation in RAW264.7 cells.

### Rftn1 knockout counteracted the protective effect of norharman on mice with AP

**Fig. 8:**
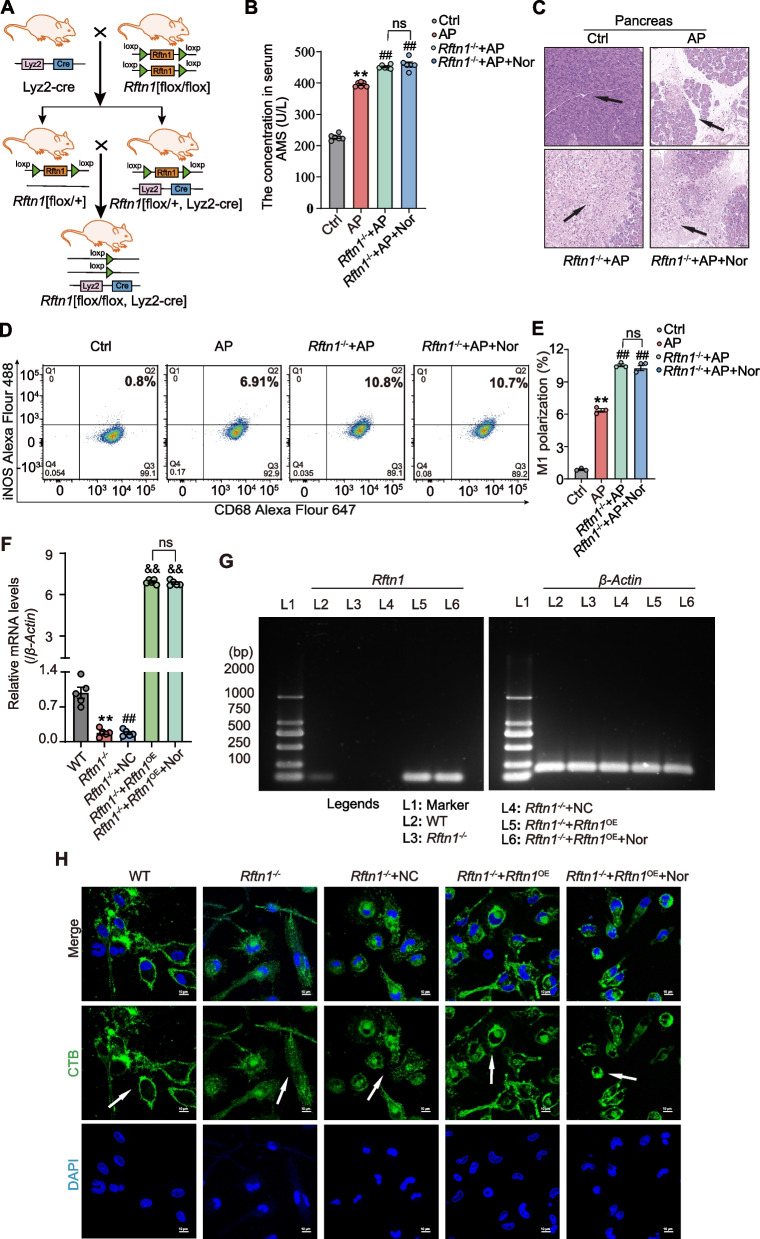
*Rftn1* knockout counteracted the protective effect of norharman on mice with AP. **A** Schematic diagram of *Rftn1* knockout mice with the genotype *Rftn1*[flox/flox, lyz2-cre]. **B** By detecting the concentrations of AMS in serum, the level of serum amylase was upregulated in the *Rftn1*^*−/−*^  + AP group but unchanged in the *Rftn1*^*−/−*^  + AP + Nor group (*n* = 6). (C) Severe pathological changes of pancreas in *Rftn1*^*−/−*^ + AP mice compared with that in AP mice (n = 5). (D-E) Compared with AP group, elevated M1 macrophage ratio was found in the spleen from *Rftn1*^*−/−*^ + AP mice, but without change after norharman treatment by flow cytometry analysis (*n* = 3). **F**, **G** Through qPCR and agarose gel electrophoresis, the effect of norharman promoting *Rftn1* expression disappeared after substitution the promoter by infection with the *Rftn1* lentiviral vector (*n* = 5). **H** Alteration of the lipid rafts structure after *Rftn1* knockout and overexpression by laser confocal microscopy (1000 ×). Data are presented as the mean ± SEM; **P < 0.01 vs. the Ctrl group or the WT group; ^##^*P* < 0.01 vs. the AP group or the *Rftn1*^*−*/*−*^ group; ^&&^*P* < 0.01 vs. the *Rftn1*^*−*/*−*^  + NC group

Given the crucial role of *Rftn1* in M1 macrophages aggravating AP, myeloid-specific *Rftn1* knockout mice were constructed (Fig. 8A, Additional file 1: Fig. S6). The concentrations of AMS in serum were significantly higher in the AP group than in the Ctrl group and markedly increased in the *Rftn1*^*−/−*^ + AP group but showed no change after norharman treatment in the *Rftn1*^*−/−*^ + AP + Nor group (Fig. 8B). Relative to the mice with AP, the *Rftn1*^*−/−*^ + AP mice showed more severe AP, as evidenced by aggravated acinar edema and necrosis in the pancreas (Fig. 8C) and an elevated M1 macrophage ratio in the spleen (Fig. 8D, E). However, these indexes were not changed after norharman treatment. These results confirmed that norharman ameliorated the pathology of AP partly by directly targeting *Rftn1* in mice.

To verify the necessity of norharman-increased H3K9/14ac binding at the promoter of *Rftn1*, we used substitution promoters by infection with *Rftn1* lentiviral vectors. Recombinant *Rftn1* lentiviral vectors contained the CMV promoter, which was completely different from the *Rftn1* self-contained promoter. Bone marrow-derived macrophages (BMDMs) were isolated and cultured for further investigation. *Rftn1* expression in BMDMs was hard to detect in the *Rftn1*^*−/−*^ group compared with the WT group, while *Rftn1* was overexpressed after recombinant *Rftn1* lentiviral vector infection in the *Rftn1*^*−/−*^  + *Rftn1*^*OE*^ group, but no significant change was observed after norharman treatment in the *Rftn1*^*−/−*^  + *Rftn1*^*OE*^ + norharman group by using qPCR and agarose gel electrophoresis (Fig. 8F, G). In addition, obvious destruction of lipid rafts structures of BMDMs was observed by laser confocal microscopy in the *Rftn1*^*−/−*^ group compared with the WT group; this parameter was rescued in the *Rftn1*^*−/−*^  + *Rftn1*^*OE*^ group, while no lipid rafts structures were observed after norharman treatment (Fig. 8H). Together, these data verified that norharman targeted HDAC1-4 to increase H3K9 acetylation depending on the promoter region of *Rftn1*, which promoted *Rftn1* transcription.

## Discussion

Our study addresses a long-standing knowledge gap about the underlying mechanism of the interaction between the gut microbiota dysfunction and metabolic imbalance in AP. Altered gut microbiota is attributed to secondary infection, which is associated with the severity of AP [36]. Moreover, metabolites produced by the gut microbial community are believed to regulate disease progression in AP [37]. Previous research has illustrated the dynamic phenotype and function of macrophages during AP development [14]. Through their important roles in the innate immune system, M1 macrophages orchestrate the proinflammatory phase of AP [16, 38]. Upon AP, bone marrow-derived and tissue-resident macrophages are recruited and differentiate into mature macrophages to infiltrate into the injured pancreas in response to the local milieu and drive T-cell-dominated adaptive immune responses [12, 39]. The phenotypic polarization and immune function of macrophages are affected by alterations in intracellular and extracellular metabolites [40]. These findings suggested that impaired pancreatic exocrine function was associated with disorders in gut microbiota composition and diversity and the crosstalk between the gut microbiota and the host immune system regulates the severity of AP. However, research on the regulation of macrophage polarization by gut microbes and their metabolites is still in its infancy. Correspondingly, we performed microbiological combined with metabolomic techniques and found that AP could induce gut microbiota dysfunction and metabolic imbalance, leading to the suppression of *Lactobacillus*-mediated tryptophan metabolism pathway.

It is extremely challenging to explore the interaction between gut microbiota dysfunction and metabolic imbalance in AP aggravation. We used LC–MS/MS to screen *Lactobacillus* metabolites of tryptophan and found that the enterobacterial metabolite norharman, which was partly exogenous and generated from tryptophan metabolism regulated by *Lactobacillus*, showed the strongest inhibitory effect on M1 macrophage activation. As a neuroactive β-carboline, norharman is a pyridoindole alkaloid that is naturally occurring, is plant-derived or in thermally processed foods, and is formed by the condensation of indoleamine (such as tryptamine) and formaldehyde, showing antioxidant, anti-inflammatory, and antitumor activities [41, 42]. Hereafter, in vitro experiments confirmed that norharman could decrease the ratio of M1 macrophages in the pancreas and spleen to ameliorate tissue lesions of the pancreas and decrease the levels of serum enzymes and inflammatory factors (*Tnf-α* and *Il-1β*) in mice with AP. We reported for the first time that norharman showed a remarkable inhibitory effect on macrophage M1 activation to alleviate AP.

Therefore, gaining a better understanding of how norharman inhibits M1 macrophage activation is urgently needed. We performed RNA sequencing and found that norharman targeted *Rftn1* to restrain M1 macrophage activation. *Rftn1* encodes the Raftlin protein, influencing the formation and maintenance of lipid rafts [24, 25]. A prospective study showed that the level of Raftlin in blood was associated with the severity of sepsis [43]. Previous studies have shown that Raftlin functions in a cell type-dependent manner. Raftlin mediates BCR and TCR signaling transduction in B and T cells [44, 45]. In endothelial cells, Raftlin can be recruited by neuropilin-1 to control intracellular trafficking of the activated vascular endothelial growth factor receptor-2 complex, which regulates proangiogenic signaling [46]. To the best of our knowledge, this is the first study to verify that norharman targets *Rftn1* to restrain the M1 polarization of macrophages.

Lipid rafts consist of sphingolipids, cholesterol, and proteins, which are cholesterol-rich and sphingomyelin-rich membrane domains that function as platforms in membrane signaling and trafficking [47]. The integrity of membrane lipid rafts was tested by utilizing fluorescently tagged CTB. In our study, *Rftn1* knockdown induced the destruction of lipid rafts structures and lipid metabolic disorder, inducing the activation of various proinflammatory responses. This phenotype could be reversed after norharman treatment. These findings indicated that norharman could promote *Rftn1* expression to suppress M1 macrophage polarization and restore the balance of lipid metabolism, which blocked various anti-inflammatory responses to alleviate AP progression.

Mechanistically, we conducted dual-luciferase reporter assays and found that norharman did not promote *Rftn1* transcription by directly binding to its promoter region, indicating other unknown regulatory mechanisms between norharman and *Rftn1* transcription. Based on the results of the literature review and molecular docking analysis, a ChIP assay was performed to confirm the interaction of HDAC1-4 and norharman. Our results showed that H3K9/14 acetylation was recruited directly to the *Rftn1* promoter region, and norharman increased the enrichment of H3K9 acetylation but not H3K14 acetylation binding to the *Rftn1* promoter region. Histone modifications mediate macrophage immune function by promoting inflammatory or polarizing gene promoters accessible to transcriptional complexes [48]. Acetylated H3K9 and H3K14 were associated with active gene expression, while deacetylation was correlated with gene repression [49]. Thus, epigenetic modification is involved in the norharman promotion of Rftn1 transcription, inhibiting pancreatic pathogenic processes.

In particular, to further validate the role of *Rftn1* in vivo, we generated Rftn1^−/−^ mice. We found that *Rftn1* knockout counteracted the protective effect of norharman in the mice with AP, suggesting that norharman ameliorates pathology in the mice with AP mainly by directly targeting *Rftn1*. In vitro, the ability of norharman to promote Rftn1 expression and lipid rafts integrity disappeared after substitution of the promoter by infection with the *Rftn1* lentiviral vector, suggesting that the promotion of *Rftn1* expression by norharman depended on the promoter of *Rftn1*.

## Conclusions

In conclusion, our study revealed that AP induced gut microbiota dysfunction and metabolic imbalance, resulting in the suppression of *Lactobacillus-*mediated tryptophan metabolism pathway. *Lactobacillus* can metabolize tryptophan into multiple functional small molecules. The enterobacterial metabolite norharman promoted *Rftn1* transcription to inhibit M1 macrophage polarization and restored lipid metabolism dysfunction, which blocked various anti-inflammatory responses to alleviate AP aggravation. The underlying mechanism by which norharman promotes *Rftn1* transcription is attributed to the inhibition of HDAC1-4 to increase H3K9 acetylation. Our findings revealed the previously unknown critical effect of the enterobacterial metabolite norharman on M1 macrophage activation, highlighted *Rftn1* as an important downstream target of norharman, and identified epigenetic modification involving the mechanism by which norharman promotes *Rftn1* expression, suggesting that norharman treatment could be a putative pharmacological intervention strategy to regulate the immune response to hinder aggravation of pancreatic inflammation.

### Supplementary Information

**Additional file 1: Table S1.** Primer sequences used for CHIP-qPCR assay. **Table S2.** Primer sequences used for qPCR assay. **Fig. S1.** The dose validation of tryptophan, LPS, and metabolites. **Fig. S2.** Toxicological detection of pancreatic toxicity, hepatotoxicity and nephrotoxicity at increasing doses of norharman. **Fig. S3.** The efficiency of si*Rftn1*/sh*Rftn1*. **Fig. S4.** The change of intestinal tight junction proteins and protective mucins. **Fig. S5.** Alteration of lipid metabolism-related genes after si*Rftn1* knockdown and norharman treatment. **Fig. S6.** PCR identification of homozygous and WT *Rtfn1*−/− mice.

**Additional file 2.** The original blots of Fig. 4I and Fig. 7B.

## Data Availability

The metabolomics data reported in this paper have been deposited in the OMIX, China National Center for Bioinformation/Beijing Institute of Genomics, Chinese Academy of Sciences (https://ngdc.cncb.ac.cn/omix, accession no. OMIX004546, OMIX004547, and OMIX004542). The data of 16S rDNA sequencing reported in this paper have been deposited in the SRA database (https://www.ncbi.nlm.nih.gov/sra/PRJNA995547, BioProject ID: PRJNA995547). The data of RNA sequencing reported in this paper have been deposited in the SRA database (http://www.ncbi.nlm.nih.gov/bioproject/995915, BioProject ID: PRJNA995915; and http://www.ncbi.nlm.nih.gov/bioproject/996232, BioProject ID: PRJNA996232).

## Abbreviations

*Acaca*: Acetyl coenzyme A carboxylase alpha
*Acacb*: Acetyl coenzyme A carboxylase beta
AP: Acute pancreatitis
BMDMs: Bone marrow-derived macrophages
CARS: Compensatory anti-inflammatory response syndrome
*Cbll1*: Casitas B-lineage proto-oncogene like 1
*Ccl2*: Chemokine (C–C motif) ligand 2
*Ccl6*: Chemokine (C–C motif) ligand 6
CHIP: Chromatin immunoprecipitation
*Cpt1a*: Carnitine palmitoyltransferase 1a
*Cpt1b*: Carnitine palmitoyltransferase 1b
*Cxcl2*: C-X-C motif ligand 2
DEGs: Differentially expressed genes
*Fabp4*: Fatty acid binding protein 4
*Fas*: Fatty acid synthase
*Fatp1*: Fatty acid transport protein 1
H3K9/14: Histone H3 at lysine 9/14
HDACs: Histone deacetylases
*Hist*: Histone cluster 1
*Il-19*: Interleukin-19
*Il-1β*: Interleukin-1beta
*Il23a*: Interleukin-23a
*Il4ra*: Interleukin 4 receptor alpha
*Il-6*: Interleukin-6
iNOS: Inducible nitric oxide synthase
*Insig*: Insulin-induced gene
*Jak2*: Janus kinase 2
*Lipg*: Endothelial lipase
LC-MS: Liquid chromatography-tandem mass spectrometry
*Lpl*: Lipoprotein lipase
LPS: Lipopolysaccharide
*Myd88*: Myeloid differentiation primary response 88
*Nfkbia*: NF-kappaB
PC: Phosphatidylcholine
*Plet1*: Placenta-expressed transcript 1
*Rftn1*: Raftlin 1
SIRS: Systemic inflammatory response syndrome
*Ski*: Ski sarcoma viral oncogene homolog (avian)
SM: Sphingomyelin
*Stat1*: Signal transducer and activator of transcription 1
*Stat2*: Signal transducer and activator of transcription 2
*Tha1*: Threonine aldolase 1
*Timp1*: Tissue inhibitor of metalloproteinase 1
*Tlr2*: Toll-like receptor 2
*Tnf-α*: Tumor necrosis factor -alpha
