# Supplementary material for: Tryptophan metabolite norharman secreted by cultivated Lactobacillus attenuates acute pancreatitis as an antagonist of histone deacetylases
Source: BMC Med. 2023 Aug 28;21:329. doi: 10.1186/s12916-023-02997-2 (PMC10463520; doi:10.1186/s12916-023-02997-2)
Supplement: Supplementary file 1 — Additional file 1: Table S1. Primer sequences used for CHIP-qPCR assay. Table S2. Primer sequences used for qPCR assay. Fig. S1. The dose validation of tryptophan, LPS, and metabolites. Fig. S2. Toxicological detection of pancreatic toxicity, hepatotoxicity and nephrotoxicity at increasing doses of norharman. Fig. S3. The efficiency of siRftn1/shRftn1. Fig. S4. The change of intestinal tight junction proteins and protective mucins. Fig. S5. Alteration of lipid metabolism-related genes after siRftn1 knockdown and norharman treatment. Fig. S6. PCR identification of homozygous and WT Rtfn1−/− mice. [file 12916_2023_2997_MOESM1_ESM.docx]

**Supplementary** **Informations**

**Tryptophan Metabolite Norharman Secreted by Cultivated *Lactobacillus* Attenuates Acute Pancreatitis as an Antagonist of Histone Deacetylases**

Qi Zhou^1,2*^, Xufeng Tao^3*^, Fangyue Guo^1,2^, Yu Wu^3^, Dawei Deng^1,4^, Linlin Lv^3^, Deshi Dong^3^, Dong Shang^1,2,4#^, Hong Xiang^1#^

^1^ Laboratory of Integrative Medicine, First Affiliated Hospital of Dalian Medical University, Dalian, 116011, China.

^2^ Institute (College) of Integrative Medicine, Dalian Medical University, Dalian, 116011, China.

^3^ Department of Pharmacy, First Affiliated Hospital of Dalian Medical University, Dalian, 116011, China.

^4^ Department of General Surgery, First Affiliated Hospital of Dalian Medical University, Dalian, 116011, China.

^*^These authors contributed equally to this work.

^#^Correspondence:

Dong Shang, Department of General Surgery, First Affiliated Hospital of Dalian Medical University, No.222 Zhongshan Road, Dalian, 116011, China. E-mail: [shangdong@dmu.edu.cn](mailto:xianghong@dmu.edu.cn/shangdong@dmu.edu.cn).

Hong Xiang, Laboratory of Integrative Medicine, First Affiliated Hospital of Dalian Medical University, No.222 Zhongshan Road, Dalian, 116011, China. E-mail: [xianghong@dmu.edu.cn](mailto:xianghong@dmu.edu.cn/shn), Tel: +86-411-83635963, Fax: +86-411-8362284

**AP model establishment**

All animals were reared in independently vented cage, and maintained under a 12 h light-dark cycle and had free access to standard laboratory feed and water. Briefly, rats with a body weight of 200-220 g were induced by retrograde injection of 5.0% sodium taurocholate (0.1 ml/100 g body weight) into the biliopancreatic duct after anesthetization with ether. For the control group, the pancreas was gently turned over. Fresh stool samples were obtained in sterile conditions after 24 h of duct infusion for 16S rDNA and untargeted metabolomic analysis.

To evaluate the toxicology of norharman, we randomly divided the mice (7–8 weeks old) with a bodyweight of 22-24 g into the control and norharman groups. Norharman was dissolved in 0.5% CMC-Na solution and given orally to the mice (1-100 mg/kg for 24 h). The levels of amylase (AMS), alanine aminotransferase (ALT), aspartate aminotransferase (AST), creatinine (CRE) and urea nitrogen (BUN) in serum were assayed by using kits. For model establishment, the animals were divided randomly into 3 groups (n=5). In the control group, the mice were injected with normal saline (50 μg/kg). The AP and norharman treatment groups were induced by the intraperitoneal injection of cerulein (50 μg/kg, MedChemExpress, Shanghai, China) 7 times (1 time per hour) and LPS (10 mg/kg) for the final injection. Based on the maximum nontoxic dose *in vivo,* norharman was given orally to mice at a dose of 100 mg/kg 3 times for 24 hours. After sacrifice, the blood pancreas, intestine, and spleen samples were harvested. The spleens were ground into single cells for flow cytometry. Parts of the pancreas and intestine were quickly frozen in liquid nitrogen for PCR and WB, and others were stored in 10% neutral buffered formaldehyde solution for HE and IF staining.

**Bacterial culture**

Wild-type (WT) *L. oris*, *L. crispatus* *and L. helveticus* were purchased from Bena Culture Collection (Beijing, China). The sterile microaerophilic environment was established by microaerobic bags (Bena culture collection, Beijing, China). Bacterial strains were cultured in MRS broth medium overnight (18 h) at 37°C for microaerophilic bacterial cultures. *Lactobacillus* strains were cultured in MRS broth medium (Solarbio, Beijing, China) with or without tryptophan (dose ranging from 0.0001 to 1%, Solarbio, Beijing, China), and proliferation was evaluated every 6 h 4 times by determining the absorbance of each well at 450 nm using a microplate reader (Bioer, China). The supernatant was collected for liquid chromatography-tandem mass spectrometry (LC-MS/MS) analysis.

**LC-MS/MS analysis**

Human serum, culture supernatant and stool samples were analyzed by LC-MS (Thermo, Ultimate 3000LC, Q Exactive HF). Q-Exactive HF (Thermo) with a flow rate of 300 μL/min was used. Compounds were separated on a Zorbax Eclipse C18 system (1.8 μm*2.1*100 mm). A 25 min linear gradient was applied from 5 to 95% acetonitrile in 0.1% formic acid. The linear gradient was as follows: 0-2 min (A: B)=95:5; 0-6 min (A: B)= 70:30; 6-7 min (A: B)=70:30; 7-12 min (A: B)=22:78; 12-14 min (A: B)=22:78; 14-17 min (A: B)=5:95; 17-20 min (A: B)=5:95; 20-21 min (A: B)=95:5; 21-25 min (A: B)=95:5. The MS instrument was operated in both positive and negative ion modes. Survey MS scans (from m/z 100-1500) were acquired in the orbitrap analyzer with resolution R=120 000 at m/z 400. Compound Discover 3.1 was used to predict metabolite information of parent compounds after conversion reactions.

**Cell viability and cytotoxicity**

RAW264.7 cells purchased from ATCC were maintained in DMEM with 10% fetal bovine serum in a humidified atmosphere of 5% CO_2_ at 37°C. Cells were seeded into 96-well plates at a density of 1×10^5^ cells/mL for 24 h and were then challenged with gradient concentrations of LPS (100 ng/mL, 1 μg/mL, 10 μg/mL, Sigma-Aldrich Co., St Louis, USA) for 1 h or 5 metabolites (0.001, 0.01, 0.1, 1, 10 µM) for 24 h: norharman, tryptophan, 5-hydroxytryptophan, indole-3-lactic acid, and indole-3-acrylic acid (Aladdin, Shanghai, China). The treated cells were incubated with Cell Counting Kit-8 (CCK-8) reagent for 1-4 h, and cell viability was assessed with an absorbance of 450 nm using a microplate reader (Bioer, China) to determine the most suitable concentration of LPS and the cytotoxicity of 7 metabolites.

**Cell transfection**

All *Rftn1* siRNAs and shRNAs were designed and synthesized by GenePharma (Shanghai, China). For transient transfection, siRNAs and shRNAs of *Rftn1* were transfected into cells according to the manufacturer's instructions.

**RNA sequencing**

RAW264.7 cells were treated with LPS alone or in combination with norharman (10 μM) for 24 h. Total RNA was extracted by using TRIzol Reagent according the manufacturer’s instructions (AG, China), and genomic DNA was removed from the preparation using DNase I (AG, China). Library preparation and sequencing were performed by Sangon Biotech (Shanghai, China) on a BGI MGI2000 sequencer. RNA sequencing was also applied to detect the differentially expressed genes (DEGs) in Rftn1 knockdown RAW264.7 cells by sh*Rftn1*.

**Confocal microscopy**

RAW264.7 cells and bone marrow-derived macrophages (1×10^5^ cells/well) were plated on micro coverglasses in 12-well plates. After transfection for 48 h, the cells were washed 3 times with PBS. Cells were incubated with Alexa Fluor 488-CTXB (5 µg/ml) for 10 min at room temperature. Then, the cells were fixed with 4% paraformaldehyde for 30 min, incubated with phalloidin-Alexa Flour 594 for 60 min, washed 3 times with PBS, and stained with DAPI. After washing, microcoverglasses were added to a 50 µl of anti-fluorescence quencher and finally visualized with a Nikon Eclipse Ti microscope (Tokyo, Japan) at 600× and 1000× magnification.

**Lipid metabolomics**

A TripleTOF 5600 Plus high-resolution tandem mass spectrometer (SCIEX, Warrington, UK) was applied to explore the metabolites of all samples. Chromatographic separation was performed using an ultraperformance liquid chromatography (UPLC) system (SCIEX, UK). An ACQUITY UPLC T3 column (Waters, UK) was applied for reversed-phase separation. The TripleTOF 5600 Plus system was utilized to investigate metabolites eluted from the column.

**Luciferase assay**

The *Rftn1* promoter region was cloned into promoter luciferase constructs and inserted into the pGL3-Basic luciferase reporter vector (GenePharma). HEK293T cells were cultured in 12-well plates with or without norharman and then transfected with plasmids containing the *Rftn1* promoter or empty control plasmids using Mate Reagent (GenePharma). The luciferase activities were analyzed after 48 h using the Dual Luciferase Reporter Assay System (GenePharma, Shanghai, China) and normalized to Renilla luciferase activity.

**Molecular docking**

Molecular docking was conducted in MOE v2018.0101. The 3D structures of mouse HDAC1, HDAC2, HDAC3 and HDAC4 were built by AlphaFold. The binding site of the native ligand in the HDAC structure was set as the binding pocket for compounds. All docked poses of molecules were ranked by London dG scoring, and then, a force field refinement was executed on the top 30 poses followed by a rescoring of GBVI/WSA dG. The best ranked pose was chosen as the final binding mode. Finally, the binding mode was visualized by PyMOL (<http://www.pymol.org>).

**Chromatin immunoprecipitation (ChIP) assay**

Briefly, H3K9/14 acetylation antibodies were incubated at room temperature for 1 h to bind to the assay plate. For *in* vivo crosslinking, culture medium containing 1% formaldehyde was added and incubated at room temperature for 10 minutes on an orbital shaker (50-100 rpm). Cell lysis and DNA shearing by sonication were performed. Subsequently, protein/DNA immunoprecipitation and crosslinked DNA reversal/DNA purification were performed. Eluted purified DNA was used for qPCR.

**ELISA**

For detection of HDAC1-4 activity, total proteins were extracted by lysis buffer with PMSF: protease inhibitor phosphatase inhibitor. A BCA Protein Quantitation Assay was used to detect the protein concentrations. HDAC1-4 activity quantification (Tongwei, Shanghai, China) was performed according to the manufacturer’s instructions. Samples were added to wells and incubated with HRP-conjugated antibody reagent for 1 hour at 37°C. Then, chromogen solutions A and B were added to each well for 15 minutes at 37°C away from direct light. The color in the wells changed from blue to yellow after stop solution was added. The optical density was measured using a microplate reader within 15 min at 450 nm.

**Isolation of bone marrow-derived macrophages (BMDMs)**

After anesthesia, the tibia and femur from wild-type C57BL/6 and *Rftn1*^-/-^ mice were separated and carefully cleaned of flesh with sterile gauze. The epiphysis was cut off, and the bone marrow was collected by inserting a syringe with sterile PBS. The cells were filtered through a 70 μm stainless steel screen before being centrifuged at 800 rpm at 4°C for 5 min. After lysis of red blood cells, the cells were resuspended in DMEM containing 10% fetal bovine serum and 20 ng/mL granulocyte macrophage colony stimulating factor (GM-CSF) in a culture flask for seven days. The medium was changed every three days.

**Infection of BMDMs with recombinant *Rftn1* lentiviral vector**

A recombinant *Rftn1* lentiviral vector containing the gene encoding full-length mouse Rftn1 containing the enhanced GFP gene was purchased from H GenePharma (Shanghai, China). Recombinant *Rftn1* lentiviral vector infection of BMDMs was carried out at MOIs of 1000 for 24 h, and then, infected BMDMs were screened by puromycin (10 mg/ml) in fresh medium for 24 h. BMDMs were treated with norharman for 24 h and collected for further analysis.

**Agarose gel electrophoresis**

After PCR or qPCR reaction, DNA or cDNA samples were loaded onto 1% agarose gels, and then run on the Electrophoresis System Jim-X for 1 h. Gels were soaked in 0.5% TBE buffers with Gold View II Nuclear Staining Dyes (5000×) (Solarbio, Beijing, China). Finally, images were taken with a Gel Image System Tanon-2500B.

**Serum enzyme assays**

The concentrations of amylase and lipase in the serum were analyzed by using commercial kits purchased from Nanjing Jiancheng Bioengineering Institute (Nanjing, China), which were detected according to the manufacturer's instructions.

**Hematoxylin and eosin and double-color immunofluorescence staining**

The pancreatic and intestinal samples were fixed with 10% neutral buffered formaldehyde solution and embedded in paraffin wax. After deparaffinization and rehydration, sections (5 μm) were prepared for further detection. The slides were stained with hematoxylin and eosin (HE), and images were captured using microscopy (Olympus) at 200× magnification. Double-color immunofluorescence staining was performed with pancreatic sections. Then, the slides were treated with anti-CD68 and iNOS antibodies overnight at 4°C. The slides were then incubated with fluorescein-conjugated horseradish peroxidase (HRP) and stained with Hoechst for 10 min. Digital images were obtained using a Pannoramic MIDI scanner (3D HISTECH, Hungary).

**Flow cytometry analysis**

The fresh spleens were placed on cell mesh, ground with a syringe needle, and washed with PBS. Single cells were harvested into tubes for centrifugation for 5 min at 1000 rpm. The supernatant was discarded, and 1 ml of PBS was added for another wash. Cell pellets were fixed and permeabilized using fixation and permeabilization solution for 40 min at 4°C. Cells were stained with antibodies against CD68 and iNOS (Santa Cruz, CA, USA). Flow cytometry analysis was performed using a flow cytometer (Fortessa; BD Biosciences), and the data were analyzed using FlowJo software (Version 10.6.0; BD Life Sciences, Ashland, Ore).

**qPCR assays**

Total RNA was extracted from tissues and cells using TRIzol according to the manufacturer’s instructions (Accurate Biotechnology, China). cDNA synthesis was performed, and gene expression was measured on an ABI 7500 and Bioer system by using a SYBR Green kit (Accurate Biotechnology, China). All PCR primers were designed by Sangong (Shanghai, China) and are shown in Additional file 1: Table S2. The results were normalized to β-actin. Fold change was determined with 2^-△△Ct^ for gene expression.

**Western blotting analysis**

For protein extraction, the tissues together with grinding beads were ground in lysis buffer by a grinder (Shanghai Jingxin Industrial Development Co., Ltd.). Cells were vortexed in lysis buffer (Solarbio, China) with protease inhibitor cocktail (Solarbio, China). The protein concentration was measured using a BCA kit (Keygen, China). Equal amounts of protein were separated by SDS-PAGE (Solarbio, China) and transferred onto PVDF membranes (Millipore; MA, USA). After the membranes were blocked with 5% fat-free milk for 2 hours, they were incubated at 4°C overnight with antibodies against Raftlin (1:500, Santa Cruz, CA, USA), β-actin (1:20000, Abclonal, Wuhan, China), acetyl-histone H3 (Lys0) (1:50, Cell Signaling Technology, Boston, USA), and histone H3 (acetylK14) (1:50, Abcam, Milton, UK). The membranes were incubated with secondary antibody for 1.5 hours at room temperature and visualized with enhanced ECL using an imaging system (Tanon 4200; Shanghai, China). β-actin were used as the internal controls.

**Statistical analysis**

All of the data are presented as the mean ± SEM. GraphPad Prism 7.0 software (GraphPad, San Diego, Calif) was applied for the statistical analysis. Differences between 2 samples were analyzed by Student’s t test, whereas differences among multiple groups were compared using one-way ANOVA. *P*<0.05 or *P*<0.01 was considered statistically signiﬁcant.

**Table S1. Primer sequences used for CHIP-qPCR assay**

| Gene | **Species** | **Primers (5'-3')** |
| --- | --- | --- |
| Primer 1 | mouse | Forward: GGGGTTGAGGCTGGGTAGAT  Reverse: CTGGAACATGGCATTTGATAGACA |
| Primer 2 | mouse | Forward: ATTCGTTGCGTGCATGTCTAT  Reverse: TCGAGGGAACCTTATGCTGAC |
| Primer 3 | mouse | Forward: TTCCAGACATGAACTAGGGATAGG  Reverse: GGGATTTTCTTCCCTGTGGC |
| Primer 4 | mouse | Forward: GCGTGTAGCTCCAATGCCT  Reverse: TTTGGGCCTGTTCATCGG |
| Primer 5 | mouse | Forward: AACAGGCCCAAAGTCACCC  Reverse: ATTGGGTCATCTTAGCCTAGCA |
| Primer 6 | mouse | Forward: CTCAAGCCCTGCACGGTAA  Reverse: TCCTGATGCAATTGGGTCAT |
| Primer 7 | mouse | Forward: TTTGGATACCTGGATAAAGTCGG  Reverse: TGGACCCATTCCACAGGTG |
| Primer 8 | mouse | Forward: TTCCCACCTGTGGAATGGG  Reverse: CATGTGCAAAATATGCTGTGCT |
| Primer 9 | mouse | Forward: AGCACAGCATATTTTGCACATG  Reverse: GGATGAAAGTTTGTGCCATGC |

**Table S2. Primer sequences used for qPCR assay**

| Gene | **Species** | **Primers (5'-3')** |
| --- | --- | --- |
| *β-Actin* | mouse | Forward: CTACCTCATGAAGATCCTGACC  Reverse: CACAGCTTCTCTTTGATGTCAC |
| *Rftn1* | mouse | Forward: GCCTGACAGATGGAGTATTCAT  Reverse: GTTCCACAACAATAGCGTCATA |
| *CD86* | mouse | Forward: ACGGAGTCAATGAAGATTTCCT  Reverse: GATTCGGCTTCTTGTGACATAC |
| *iNOS* | mouse | Forward: ACTCAGCCAAGCCCTCACCTAC  Reverse: TCCAATCTCTGCCTATCCGTCTCG |
| *Tnf-α* | mouse | Forward: ATGTCTCAGCCTCTTCTCATTC  Reverse: GCTTGTCACTCGAATTTTGAGA |
| *Cbll1* | mouse | Forward: TGATGCCACCGCCTCCTCTG  Reverse: AAAGGTTTCCTGACTGACCGAACG |
| *Ccl6* | mouse | Forward: GGCTTTCAAGACACTTCTTCAG  Reverse: CCCTCCTGCTGATAAAGATGAT |
| *Hist* | mouse | Forward: GTGTGCTGAAGGTGTTCCTGGAG  Reverse: AGCCGCCGAATCCGTAGAGG |
| *Il19* | mouse | Forward: ACAGAGACAGGGTGTTCCAGGAC  Reverse: ACGCAGAGGAAAGAGTTGGCAATG |
| *Lipg* | mouse | Forward: ACAAACACCTTCCTTGTCTACA  Reverse: CAGGTAGTTGCGAAACTCATTC |
| *Plet* | mouse | Forward: GGTGGGGATGTAACCTATACAG  Reverse: CTGTCGTTGCACTTCTCATATG |
| *Ski-2* | mouse | Forward: AGTTCGACTATGCCAACAAGTA  Reverse: GTCGTCTGTTTTGGGTCTTATG |
| *Tha1* | mouse | Forward: ATCCTTCTGTTTCTCTAAGGGC  Reverse: TTGGAGTCCTTTAGCGAATCTC |
| *Timp* | mouse | Forward: GCAAAGAGCTTTCTCAAAGACC  Reverse: CTCCAGTTTGCAAGGGATAGAT |
| *Ccl2* | mouse | Forward: TTTTTGTCACCAAGCTCAAGAG  Reverse: TTCTGATCTCATTTGGTTCCGA |
| *Jak2* | mouse | Forward: ACATTCTTACCAAAGTGCGTTC  Reverse: GCTGAATGAATCTGCGAAATCT |
| *Il4ra* | mouse | Forward: GCTTTGGTATTGTGTACTCGTC  Reverse: GGATGGTGATCTGTCATCGTAG |
| *Il23a* | mouse | Forward: CAGCGGGACATATGAATCTACT  Reverse: TTGAAGATGTCAGAGTCAAGCA |
| *Nfkbia* | mouse | Forward: CTGGTTTCGCTCTTGTTGAAAT  Reverse: GGGTAGCATCTGGAGATTTTCC |
| *Stat1* | mouse | Forward: GATCCGTCAGCAGCTTAAAAAG  Reverse: CTGACAACACCTGCTTGTTTTT |
| *Stat2* | mouse | Forward: TGAGTCACATGCTTCGGTATAA  Reverse: TGAAGTAACTCCATGACTTGGG |
| *Tlr2* | mouse | Forward: GACTCTTCACTTAAGCGAGTCT  Reverse: AACCTGGCCAAGTTAGTATCTC |
| *Acaca* | mouse | Forward: CCCAGAGATGTTTCGGCAGTCAC  Reverse: GTCAGGATGTCGGAAGGCAAAGG |
| *Acacb* | mouse | Forward: AGATCGCCTCCACCATCGTAGC  Reverse: CTGTCCTCCGTCCACTCCACTG |
| *Cpt1a* | mouse | Forward: CTACATCACCCCAACCCATATT  Reverse: GATCCCAGAAGACGAATAGGTT |
| *Cpt1b* | mouse | Forward: TTCACCTGGGCTACACGGAGAC  Reverse: GCCTTGGCTACTTGGTACGAGTTC |
| *Fabp4* | mouse | Forward: CATCCGGTCAGAGAGTACTTTT  Reverse: TAGGGTTATGATGCTCTTCACC |
| *Fatp1* | mouse | Forward: CCTCTCTGTTCTGATTCGTGTT  Reverse: GTCCAGCATATACCACTACTGG |
| *Fas* | mouse | Forward: TAAAGCATGACCTCGTGATGAA  Reverse: GAAGTTCAGTGAGGCGTAGTAG |
| *Insig* | mouse | Forward: GTCACCCAACTGTTAGTCTACA  Reverse: ATTCATACATTGCCAGTTGACG |
| *Lpl* | mouse | Forward: CCTGATGACGCTGATTTTGTAG  Reverse: CAATGAAGAGATGAATGGAGCG |


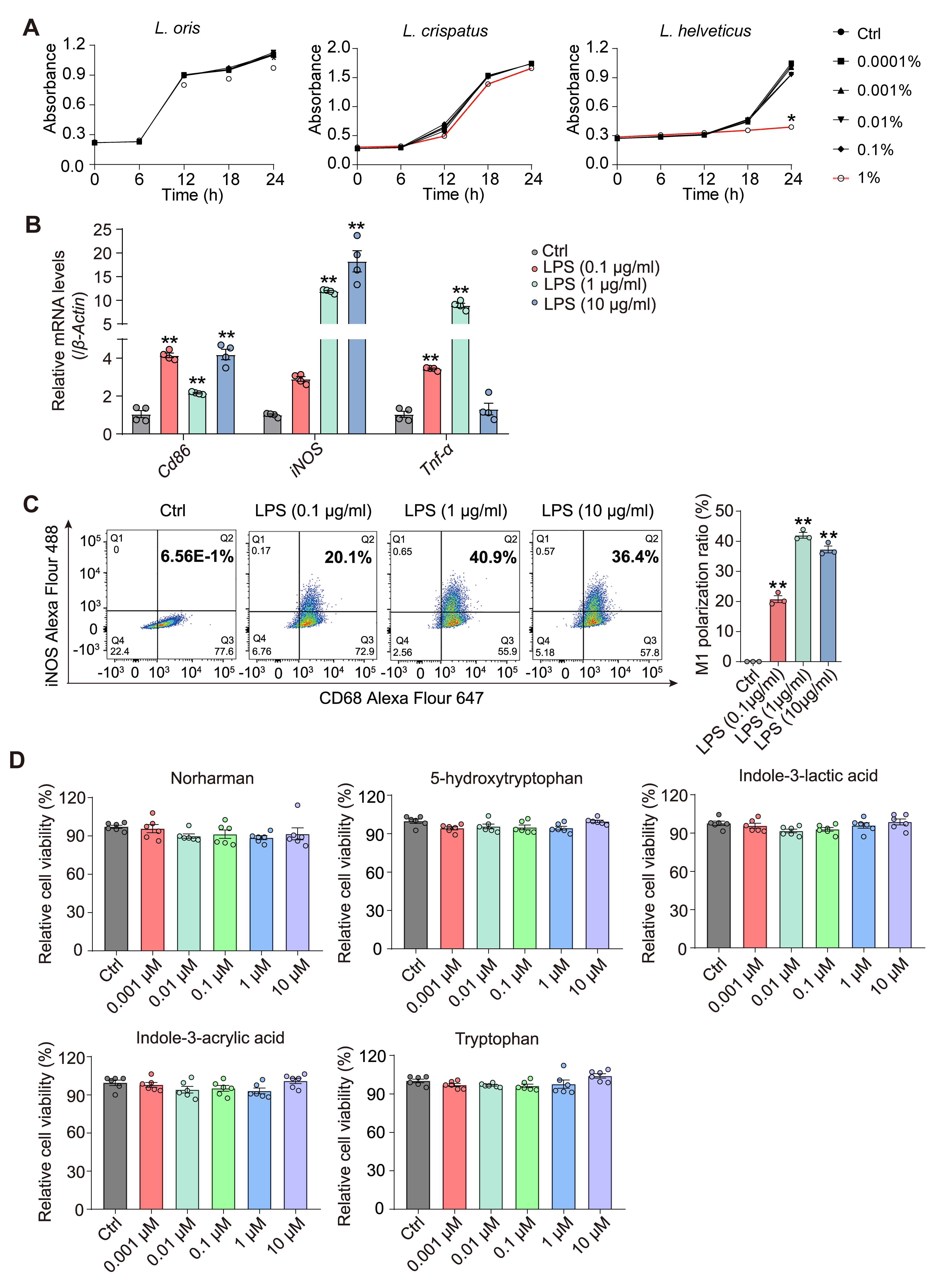


**Fig. S1. The dose validation of tryptophan, LPS, and metabolites.** (A) The effect of tryptophan on the proliferation of 3 *Lactobacillus* strains at different doses. (B) The optimal concentration for macrophage activation by qPCR (n=4). (C) The optimal concentration for M1 macrophage activation by flow cytometry analysis (n=3). (D) The cytotoxicity of 5 metabolites in RAW264.7 cells (n=6). Data are presented as the mean ± SEM; **P*<0.05 and ***P*<0.01 vs. the Ctrl group.


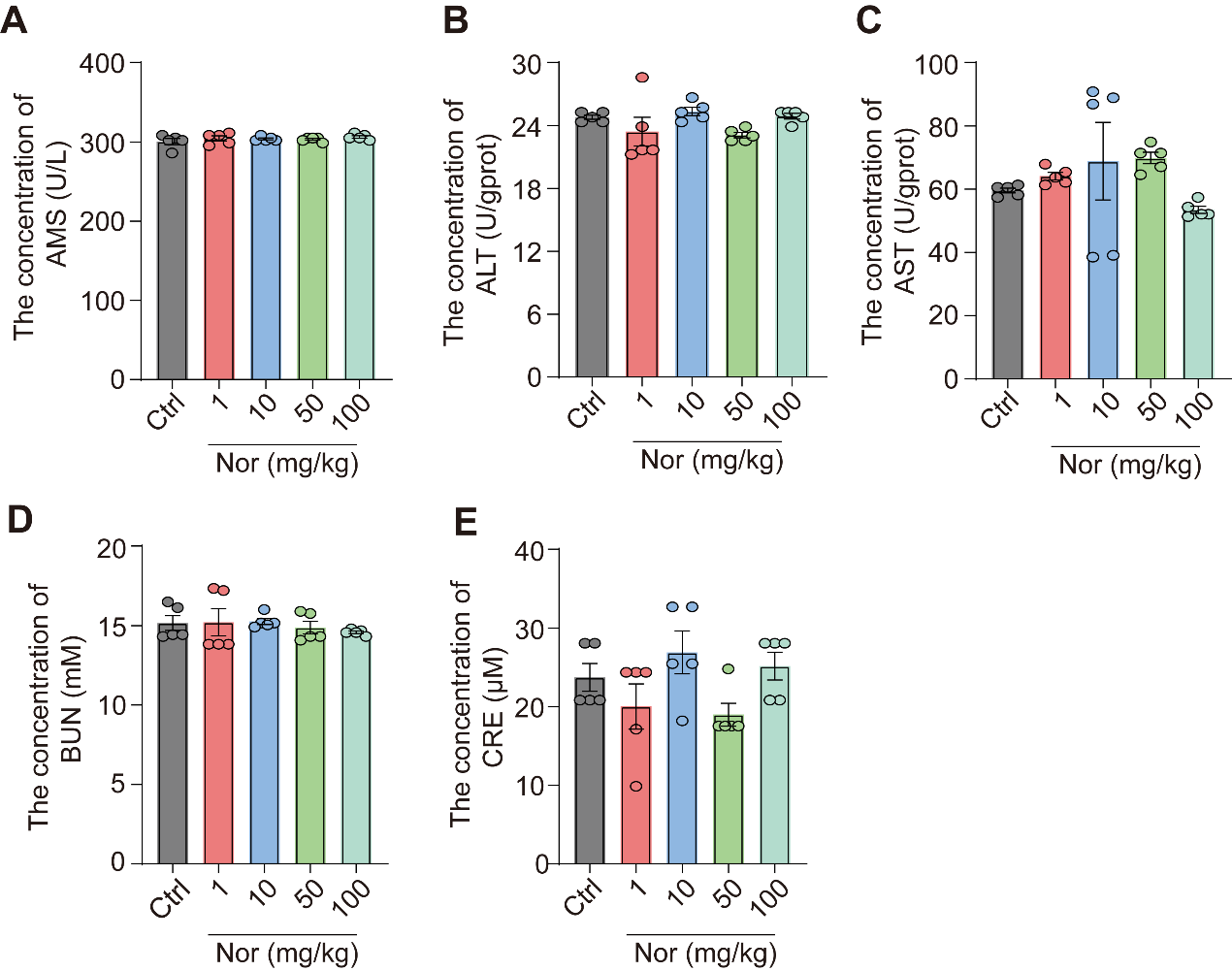


**Fig. S2.** **Toxicological detection of pancreatic toxicity, hepatotoxicity and nephrotoxicity at increasing doses of norharman.** (A) α-AMS for pancreatic toxicity. (B-C) ALT and AST for hepatotoxicity. (D-E) BUN and CRE for nephrotoxicity (n= 5). Data are presented as the mean ± SEM.


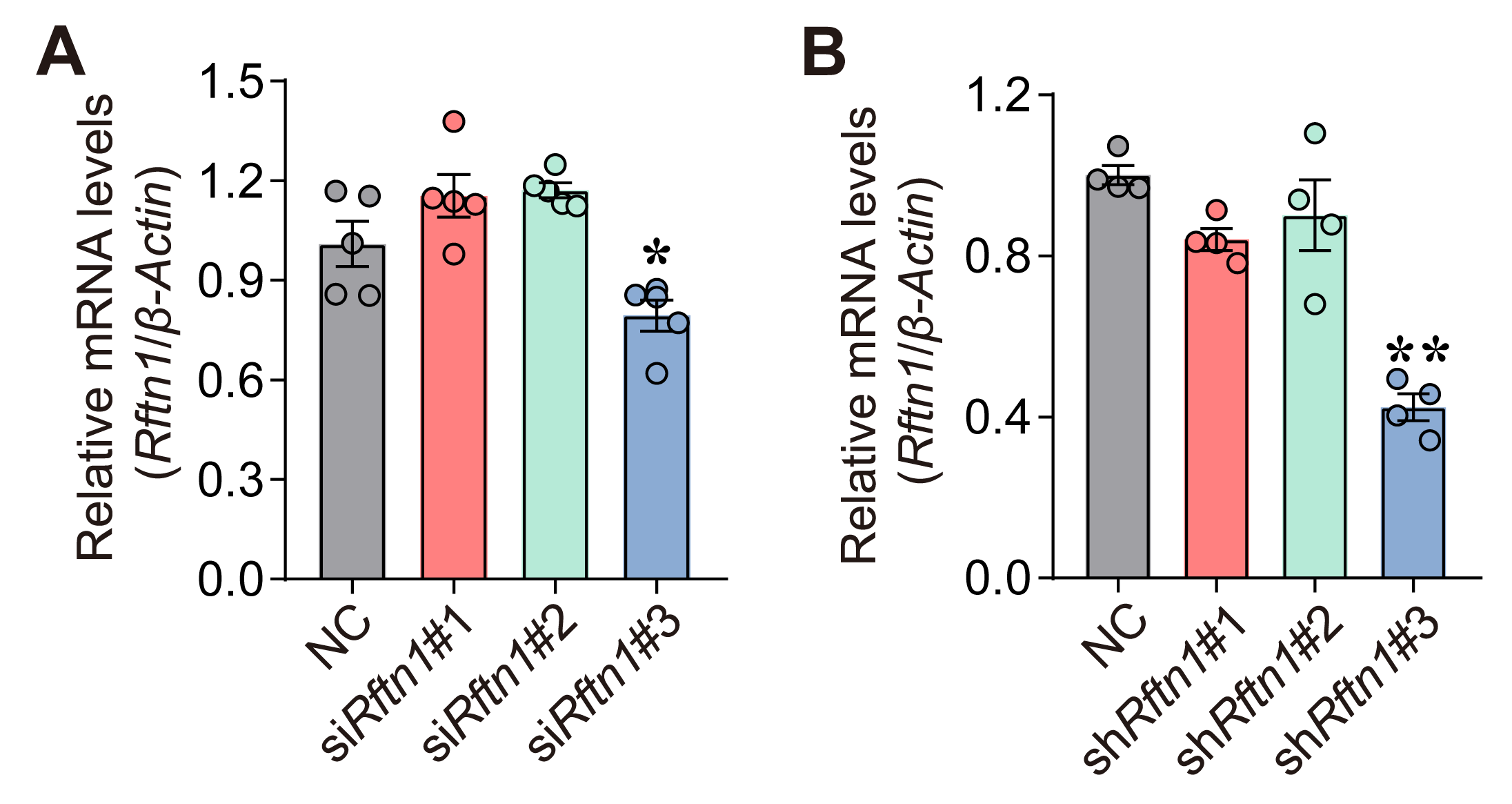


**Fig. S3. The efficiency of si*Rftn1*/sh*Rftn1*.** (A) The knockdown efficiency of three si*Rftn1*s (n=5). (B) The knockdown efficiency of three sh*Rftn1*s (n=4). Data are presented as the mean ± SEM; **P*<0.05 and ***P*<0.01 vs. the NC group.


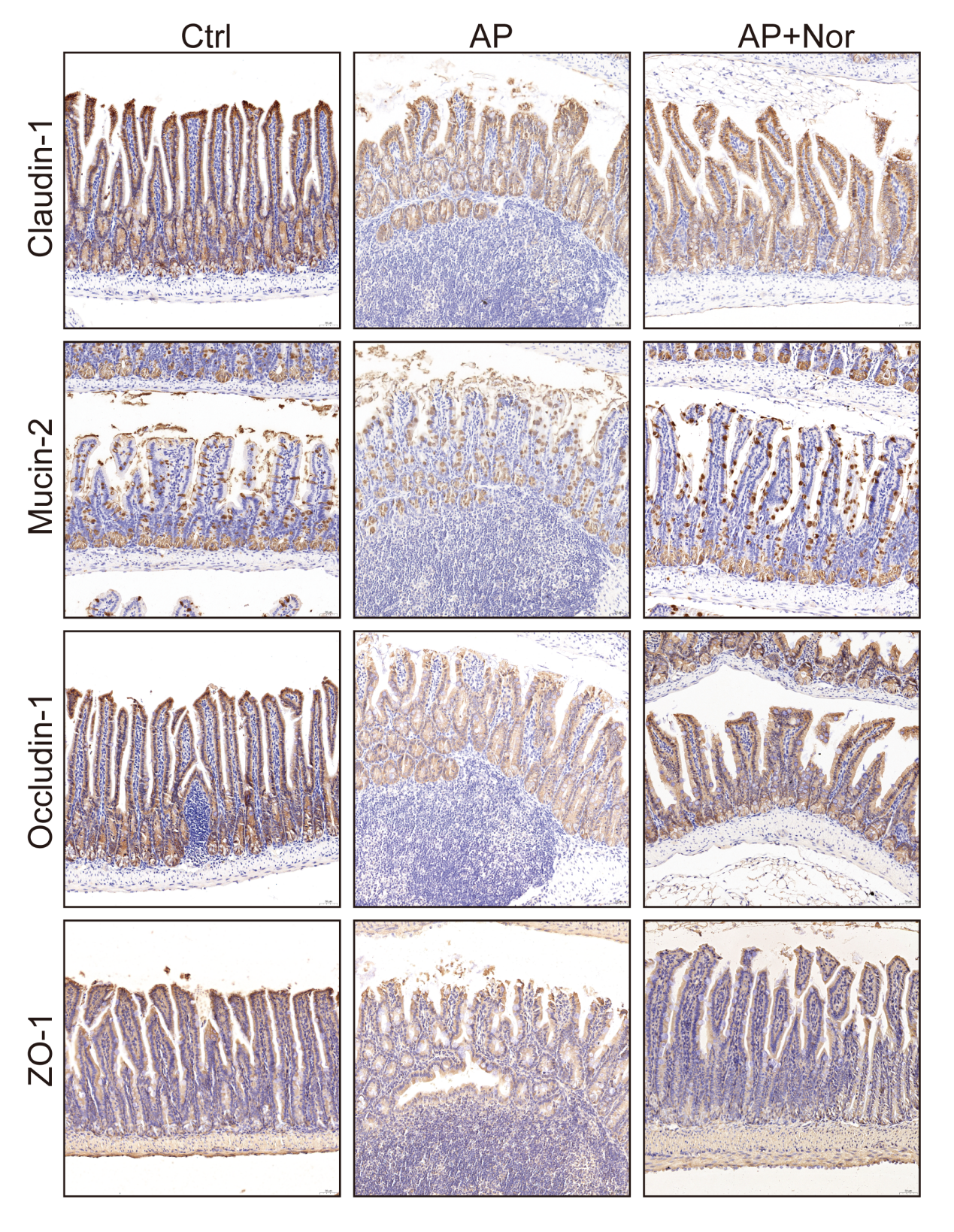


**Fig. S4. The change of intestinal tight junction proteins and protective mucins.** The expression of intestinal tight junction proteins (Claudin-1, Occludin-1 and ZO-1) and protective mucins (Mucin-2) was decreased in AP group compared with Ctrl group, but increased in AP+nor group.

**
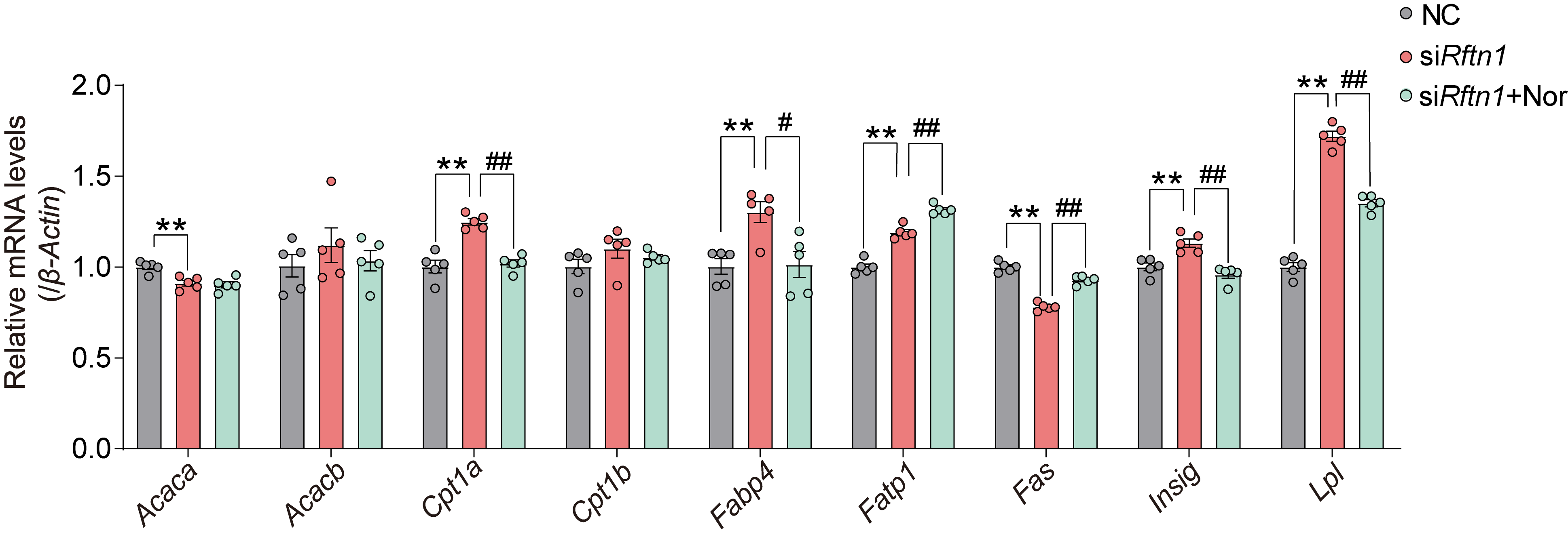
**

**Fig. S5. Alteration of** **lipid metabolism-related genes after si*Rftn1* knockdown and norharman treatment.** Data are presented as the mean ± SEM (n=5); **P*<0.05 and ***P*<0.01 vs. the NC group.


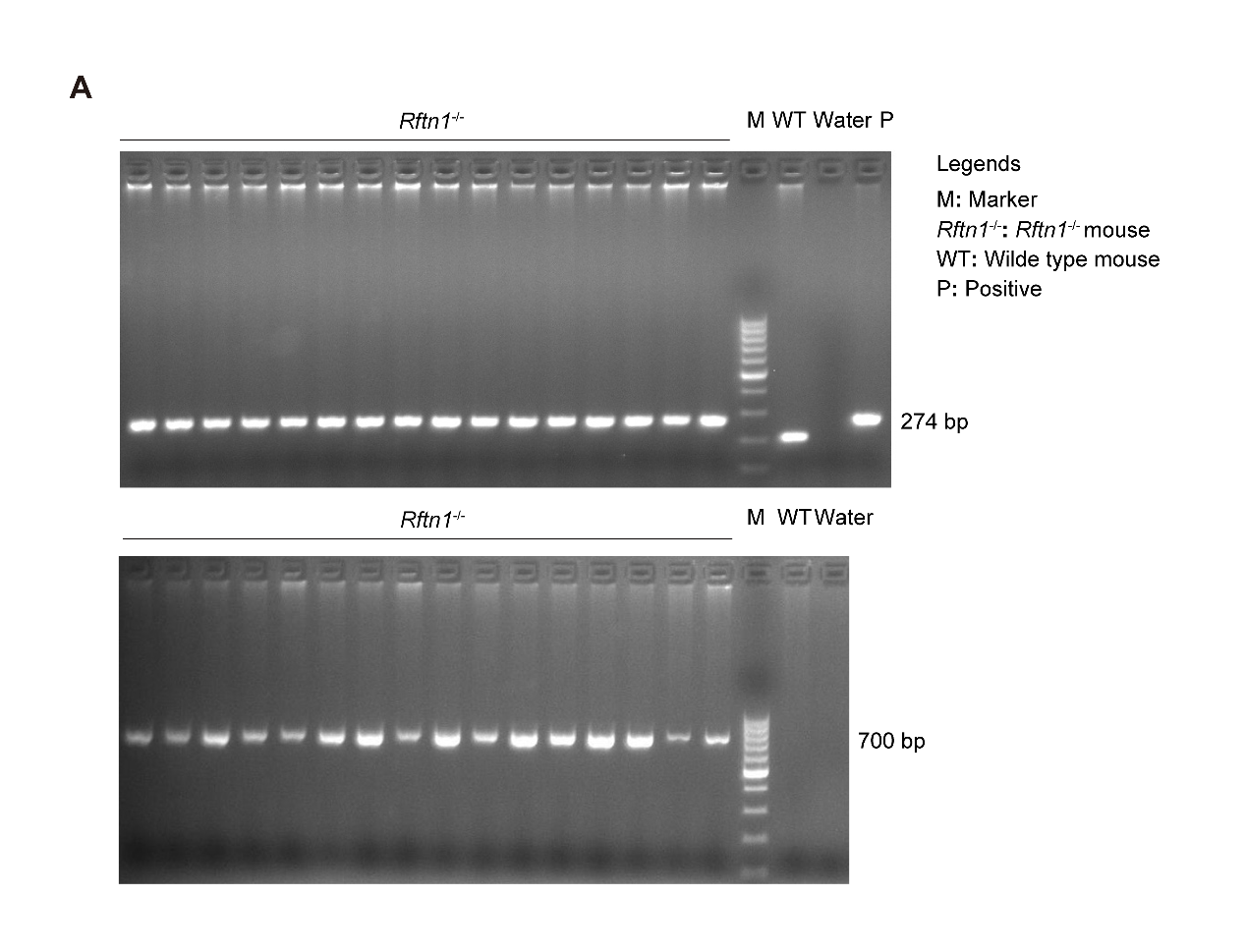


**Fig. S6. PCR identification of homozygous and WT *Rtfn1* ^-/-^ mice.**
