# Supplementary material for: Tryptophan metabolite norharman secreted by cultivated Lactobacillus attenuates acute pancreatitis as an antagonist of histone deacetylases
Source: BMC Med. 2023 Aug 28;21:329. doi: 10.1186/s12916-023-02997-2 (PMC10463520; doi:10.1186/s12916-023-02997-2)
Supplement: Supplementary file 2 — Additional file 2. The original blots of Fig. 4I and Fig. 7B. [file 12916_2023_2997_MOESM2_ESM.docx]

AP+Nor

AP

Ctrl

RFTN1

63 KD

The original blot of RFTN1 of pancreas in Fig. 4I





Ctrl

AP+Nor

AP

β-Actin

42 KD

The original blot of β-Actin of pancreas in Fig. 4I





AP+Nor

AP

Ctrl

63 KD

RFTN1

The original blot of RFTN1 of intestine in Fig. 4I





AP+Nor

AP

Ctrl

42 KD

β-Actin

The original blot of β-Actin of intestine in Fig. 4I





Nor

Ctrl

RFTN1

63 KD

The original blot of RFTN1 of cells in Fig. 7B





Nor

Ctrl

β-Actin

42 KD

The original blot of β-Actin of cells in Fig. 7B
